# CRISPR interference to interrogate genes that control biofilm formation in *Pseudomonas fluorescens*

**DOI:** 10.1038/s41598-019-52400-5

**Published:** 2019-11-04

**Authors:** Marie-Francoise Noirot-Gros, Sara Forrester, Grace Malato, Peter E. Larsen, Philippe Noirot

**Affiliations:** 1 Biosciences Division, Argonne National Laboratory, Lemont, IL60439 United States; 2 Department of Bioengineering, University of Illinois Chicago, Chicago, IL60607 United States

**Keywords:** Biofilms, Molecular biology

## Abstract

Bacterial biofilm formation involves signaling and regulatory pathways that control the transition from motile to sessile lifestyle, production of extracellular polymeric matrix, and maturation of the biofilm 3D structure. Biofilms are extensively studied because of their importance in biomedical, ecological and industrial settings. Gene inactivation is a powerful approach for functional studies but it is often labor intensive, limiting systematic gene surveys to the most tractable bacterial hosts. Here, we adapted the CRISPR interference (CRISPRi) system for use in diverse strain isolates of *P*. *fluorescens*, SBW25, WH6 and Pf0-1. We found that CRISPRi is applicable to study complex phenotypes such as cell morphology, motility and biofilm formation over extended periods of time. In SBW25, CRISPRi-mediated silencing of genes encoding the GacA/S two-component system and regulatory proteins associated with the cylic di-GMP signaling messenger produced swarming and biofilm phenotypes similar to those obtained after gene inactivation. Combined with detailed confocal microscopy of biofilms, our study also revealed novel phenotypes associated with extracellular matrix biosynthesis as well as the potent inhibition of SBW25 biofilm formation mediated by the PFLU1114 operon. We conclude that CRISPRi is a reliable and scalable approach to investigate gene networks in the diverse *P*. *fluorescens* group.

## Introduction

Biofilms are the prevalent state of bacterial life in nature^1^ and biofilm formation is an integral part of the prokaryotic life cycle. Biofilms are clusters of microorganisms embedded in a self-produced matrix of extracellular biopolymers that provide shelter, allow cooperation between bacterial cells, interactions with the environment, and confer the ability to colonize new niches by dispersal of microorganisms from the microbial clusters^2–4^. Biofilms can form on virtually any abiotic or biotic surface. Often associated with chronic infections and resistance to antibiotic treatments, they are generally considered harmful to human health^5,6^. In contrast, biofilm-forming non-pathogenic bacteria can effectively protect plants from infection by pathogens^7^, promote plant growth and stimulate symbiotic interactions between mycorrhizal fungi and plant roots^8–12^. Thus, there is considerable interest in deciphering at a molecular level the regulatory mechanisms of biofilm formation and dispersion to combat biofilms but also to better control them.

Biofilm formation is triggered by environmental cues and involves coordinated responses from a number of cellular processes such as flagellar assembly and secretion of extracellular polymeric substances (EPS). In bacteria, two-component systems (TCS) sense environmental stimuli and translate this information into cellular responses through coordinated regulation of genetic programs^13–15^. In Gram-negative bacteria, the well-characterized GacA/S TCS regulates the expression of genes involved in quorum sensing, stress responses, biofilm formation and virulence^16,17^. The GacA/S TCS is composed of a membrane-bound sensor histidine kinase GacS and its cognate response regulator GacA^16^. In γ-proteobacteria such as *Pseudomonas* and *Halomonas*, inactivation of either GacA or GacS dramatically affects the production of EPS, secondary metabolites and iron homeostasis^18–21^. EPS are mainly composed of polysaccharides such as alginate and contribute to the biofilm architecture. EPS production is regulated by the Gac/Rsm signaling cascade, involving GacA/S TCS and the non-coding small regulatory RNAs RsmZ and RsmY^17,18,22–24^. This signaling cascade regulates about 700 genes involved in a wide range of biological functions, including biofilm formation and oxidative stress response^17,24,25^.

Cyclic diguanylate (c-di-GMP), a near universal intracellular signaling messenger, regulates many aspects of bacterial growth and behavior^26,27^, including motility, virulence and the transition from motile-to-sessile lifestyle leading to biofilm formation^28,29^. Intracellular levels of c-di-GMP can be modulated through the balanced activities of two classes of enzymes: (i) the diguanylate cyclases (DGCs) that synthetize c-di-GMP from two GTPs and (ii) the diguanylate phosphodiesterases (PDEs) that breakdown c-di-GMP into pGpG and GMP^28,29^. In addition to DGCs (which contain GGDEF domains) and PDEs (which contain EAL or HY-GYP domains), bacterial genomes generally encode multiple c-di-GMP-binding proteins, including PilZ-domain effectors and proteins with ‘degenerate’ GGDEF/EAL domains that are enzymatically inactive but still bind c-di-GMP^30,31^. Although the propensity of bacteria to form biofilm correlates with higher intracellular concentrations of c-di-GMP^32^, it is not currently understood how the multiple DGCs and PDEs contribute to a specific phenotypic outcome^26^. Extensive studies in *P*. *aeruginosa* uncovered the roles of many DGCs, PDEs and c-di-GMP-binding effectors during biofilm development, from the initial stage of cell adhesion on a surface to colony formation and biofilm maturation and dispersion^28^. In *P*. *aeruginosa*, the initial biofilm formation stage is governed by the diguanylate cyclase promoting biofilm protein GcbA that regulates surface attachment via modulation of the flagellum-driven motility^33^. GcbA is also involved in biofilm dispersion through post-translational processing of the chemosensory protein BdlA, known to regulate PDE activities such as that of the DipA protein^34–36^. The PDE BifA modulates biofilm formation and motility by altering exopolysaccharide production and flagellar function^37–39^. C-di-GMP also regulates alginate synthesis through binding to the PilZ-type domain of alginate co-polymerase Alg44. Additional levels of regulation involve the PDE RimA which modulates the activity of RimK, an enzyme that modifies the ribosomal protein RpsF by adding glutamate residues to its C terminus, thus altering ribosome abundance and function^40,41^. Altogether, these studies reveal that c-di-GMP-binding enzymes and proteins act in a coordinated manner to control the various stages of the planktonic-to-biofilm transition, altering motility, promoting cell adhesion, producing EPS and shaping biofilm architecture.

Here, we focus on c-di-GMP-associated regulators that control biofilm formation in the rhizobacterium *P*. *fluorescens*. Soil and plant-associated bacteria such as *P*. *fluorescens* can provide beneficial ecological services to various plants^42–44^ and form dynamic and highly structured biofilm at aspen roots^11^. Comparative genomics of *P*. *fluorescens* strain isolates revealed a conserved core genome and a widely diverse pan-genome encoding specialized activities, many being relevant for colonization of the rhizosphere and plant-bacteria interactions^45,46^. *P*. *fluorescens* genomes typically encode about 50 proteins with c-di-GMP-binding signatures, including homologs for GbcA, BifA, DipA and Alg44. Extensive phenotypic screens have been performed in *P*. *fluorescens* to identify genes controlling biofilm formation. In *P*. *fluorescens* SBW25, screens for wrinkly spreader biofilm phenotypes, which are associated with enhanced formation of cellulose-based matrix at the air-liquid interface, identified many DGCs and associated regulators involved in c-di-GMP homeostasis^47–51^. In *P*. *fluorescens* Pf0-1, a systematic survey of knockout mutants revealed that about a third of c-di-GMP-associated proteins exhibit strong biofilm phenotypes across many growth conditions whereas the remaining mutants exhibited weak phenotypes in only a small number of conditions^52^. However, the biological roles of most the c-di-GMP-binding proteins still remain to be characterized. Understanding these biological roles relies on our ability to associate specific c-di-GMP-related proteins with particular biofilm phenotypes. Most previous studies, however, have been based on a colorimetric assay that measures EPS accumulation in mature biofilms^53^. While this assay is rapid and reliable, it cannot report on the full range of phenotypes (e.g., cell abundance, EPS architecture) that characterize biofilm development.

In this work, we used a CRISPRi-based approach to investigate the role of genes belonging to the c-di-GMP regulatory network in biofilm formation and architecture. CRISPRi can be used to modulate gene expression and to study genes essential for cell survival^54–56^. We adapted the CRISPRi system to *P*. *fluorescens* and validated its application for gene silencing in SBW25, WH6 and Pf0-1 strains. In SBW25, we applied our CRISPRi system to downregulate genes involved in c-di-GMP signaling pathways allowing the quantitative study of phenotypes at cell, colony and biofilm levels. CRISPRi-mediated gene silencing is a robust and reliable approach for quantitative phenotyping of complex bacterial traits such as swarming motility and biofilm mass, structure and composition that can be used to discover the function of uncharacterized genes.

## Results

### Design and validation of CRISPRi in *P*. *fluorescens*

In the CRISPRi system, a small guide RNA (gRNA) directs the catalytically inactive dCas9 protein to bind at or near a promoter region and sterically hinder the initiation or elongation of transcription, resulting in silencing of gene expression^54,55^. CRISPRi systems have been previously shown to sterically block transcription of genes in the model bacteria *E*. *coli* and *B*. *subtilis*^55,56^. We adapted the CRISPRi system for *P*. *fluorescens* by constructing a system comprised of two compatible plasmids (Supplementary Fig. S1). One plasmid carries the *S*. *pyogenes* dCas9 gene under control of the P*tetA* promoter that can be induced by the presence of anhydrotetracyclin (aTc)^57^ in the growth medium. The other plasmid constitutively expresses a gRNA (see Materials and Methods).

**Figure 1:**
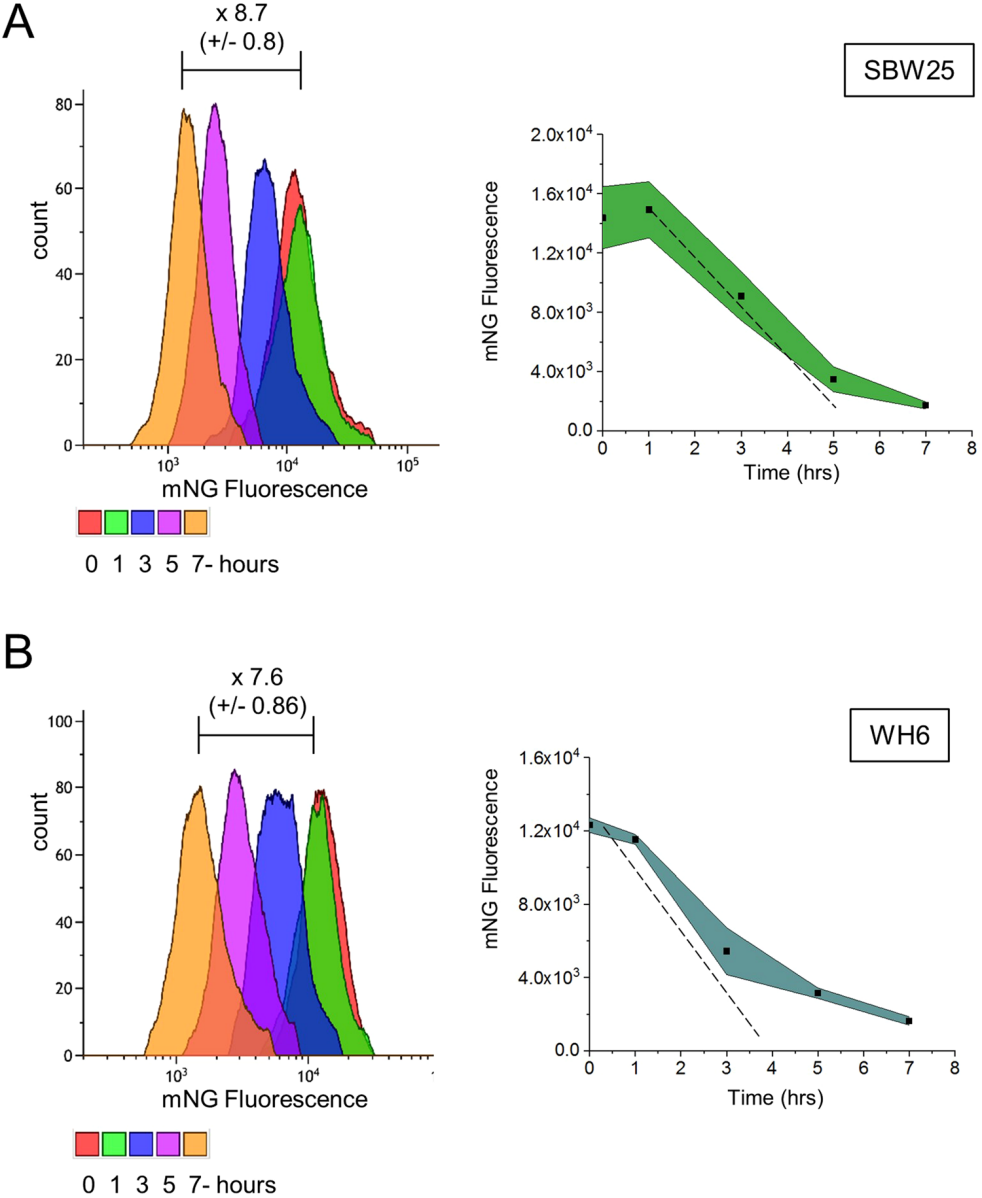
Gene silencing by the CRISPRi system in *P*. *fluorescens*. SBW25 (**A**) and WH6 (**B**) strains contain an identical DNA cassette coding for the mNG fluorescent protein expressed from a constitutive promoter (Pc) inserted at a similar chromosomal locus. Both strains harbor the pPFL-Cas9 plasmid and the pPFL-sgRNA plasmid expressing the gRNA Pc2 targeting transcription elongation of the mNG gene at a site overlapping the start of the ORF (see also Fig. S2). Green fluorescence intensities were monitored by flow cytometry over 7 hours after induction of dCas9 (illustrated by colors, right panels). Upon silencing, fluorescence intensity decreases overtime due to dilution from cell divisions (left panels). Dotted lines represent the expected dilution of the GFP in SBW25 (doubling time 135 min) and WH6 (doubling time 120 min).

We assessed the functionality of our CRISPRi system in three *P*. *fluorescens* strain isolates: SBW25, WH6 and Pf0-1. In each strain, the *mNG* gene encoding the mNeonGreen fluorescent protein, was placed under control of the constitutive Pc promoter and inserted at similar chromosomal locations in all strains^11^. We designed two pairs of gRNAs, one pair (Pc4 and Pc5) targeting transcription initiation at the Pc promoter and the other pair (Pc2 and Pc3), targeting transcription elongation at a site overlapping the start of the open reading frame (ORF, Supplementary Table S1). gRNAs target DNA sites by either copying the template (T, Pc3 and Pc4) or non-template (NT, Pc2 and Pc5) strand (Supplementary Fig. S2). Upon induction of dCas9 expression, the effect of each gRNA on fluorescence intensity was monitored over time using flow cytometry (Fig. 1, Supplementary Fig. S2). We found that gRNAs Pc4 and Pc5 targeting the transcription initiation resulted in the highest decrease of mNG fluorescence relative to the control without gRNA. However, part of this decrease was observed in the absence of inducer (T = 0), suggesting that dCas9 is expressed at some basal level. The basal expression of dCas9 had minimal effects with the Pc2 gRNA targeting transcription elongation and copying the NT strand (gRNA_NT_) in SBW25 and WH6 but not in Pf0-1. In SBW25, the downregulation of *mNG* expression depended on the dose of aTc inducer (Supplementary Fig. S2E). At the mRNA level, a dose-dependent downregulation of *mNG* was observed upon increasing aTc concentration, with a maximum 30-fold repression with 100 ng/ml of inducer (Supplementary Fig. S2F). Next, we evaluated the kinetics of fluorescence decrease caused by elongation-blocking gRNA_NT_ in SBW25 and WH6 (Fig. 1). A complete block of *mNG* expression is expected to result in a 50% decrease of the fluorescence intensity after each cell doubling, owing to the short maturation time and high stability of the mNG protein^58^. In the conditions of our assay, the generation times for SBW25 and WH6 were 135 min and 120 min, respectively. For both strains, the observed decrease of fluorescence is only slightly slower than the expected dilution of the mNG protein from cell division, indicating a strong although incomplete silencing (Fig. 1). Monitoring of the effect of targeting the T strand for elongation transcription block by Pc3 gRNA revealed a lower decrease of fluorescence (3.2-fold after 7 hours) compared to the non-induced condition (Supplementary Fig. S2, Supplementary Table S2). This result corroborates previous studies highlighting the importance of using the NT strand for maximal repression^54,55^. Based on these findings, we chose to design our next experiments using (i) gRNA_NT_ guides that target the start of ORFs and (ii) a strain harboring the two plasmids and expressing dCas9 but no gRNA as a control for no CRISPRi activity.

### CRISPRi silencing of genes involved in cytokinesis and morphogenesis

To assess the efficacy of our CRISPRi system to generate observable phenotypes in *P*. *fluorescens*, we targeted the *ftsZ* and *mreB* genes that are essential for bacterial survival and when mutated, produce characteristic defects in cell division or cell shape. Upon depletion of tubulin-like protein FtsZ, bacterial cells typically grow as long and non-septate filaments resulting from failure to assemble a functional FtsZ division ring^59–62^. The depletion of actin-like MreB in *E*. *coli* cells results in loss of the rod-shaped morphology and gives rise to enlarged round cells^63^. In *B*. *subtilis* which encodes several MreB-like proteins, depletion of each homolog results in aberrant cell morphology phenotypes such as wider, inflated and twisted cells, ultimately leading to cell lysis^64,65^.

**Figure 2:**
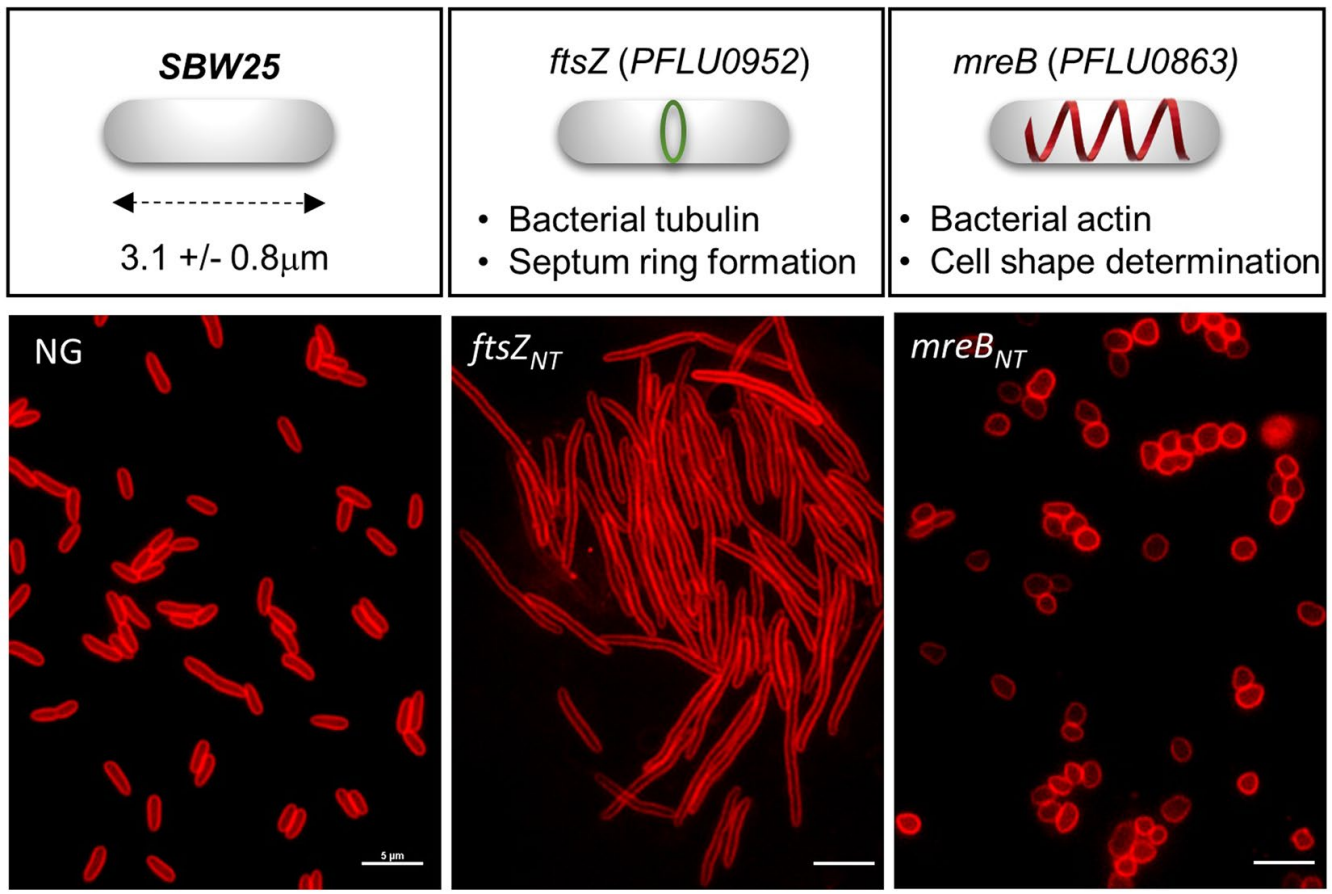
CRISPRi silencing of genes involved in cell division and cell morphology in *P*. *fluorescens*. Cells harboring the pPFL-dCas9 plasmid and the pPFL-gRNA plasmid expressing a gRNA_NT_ targeting the start of the ORFs to block transcription elongation. Control with no guide (left panel); silencing of *ftsZ* (PFLU0952) using a ftsZ_NT_ guide (center panel), and silencing of *mreB* (PFLU0863) using a mreB_NT_ guide (right panel). Strains were grown 5 hours in the presence or absence of inducer (aTc 0.1 μg/ml). Cells were stained with the membrane fluorescent dye FM 4–64 prior to observation by epifluorescence microscopy. Scale bars indicate 2 μm.

*P*. *fluorescens* SBW25 *ftsZ* (PFLU0952) and *mreB* (PFLU0863) were silenced by expressing a gRNA targeting *ftsZ* (*ftsZ*_*NT*_) or mreB (*mreB*_*NT*_) in cells induced for dCas9 expression. After 5 hours of incubation at 25 °C in the presence of inducer (aTc 100 ng/ml), cells expressing the *ftsZ*_*NT*_ guide exhibited a characteristic cell filamentation phenotype (Fig. 2) consistent with *ftsZ* knockdown. The morphological defects appeared 3 hours after induction, involved all observed cells after 5 hours, and this phenotype persisted in overnight cultures (Supplementary Fig. S4). Cells expressing the *mreB*_*NT*_ guide exhibited the characteristic morphological defects associated with a depletion of MreB, including inflated and round cells with part of them bursting in overnight cultures (Fig. 2, Supplementary Fig. S4). Of note, the expected morphological defects could also be observed using gRNA_T_ targeting the *ftsZ* and *mreB* genes when inducer concentration was 5-fold higher and incubation times were longer (Supplementary Fig. S5). These findings confirm the importance of using gRNA_NT_ for maximal repression and also indicate that a milder repression can be obtained with gRNA_T_. These results show that CRISPRi is suitable for gene silencing in *P*. *fluorescens* associated with phenotypic analyses over several hours.

### CRISPRi silencing of the two-component sensor kinase GacS impairs mobility and biofilm formation

**Figure 3:**
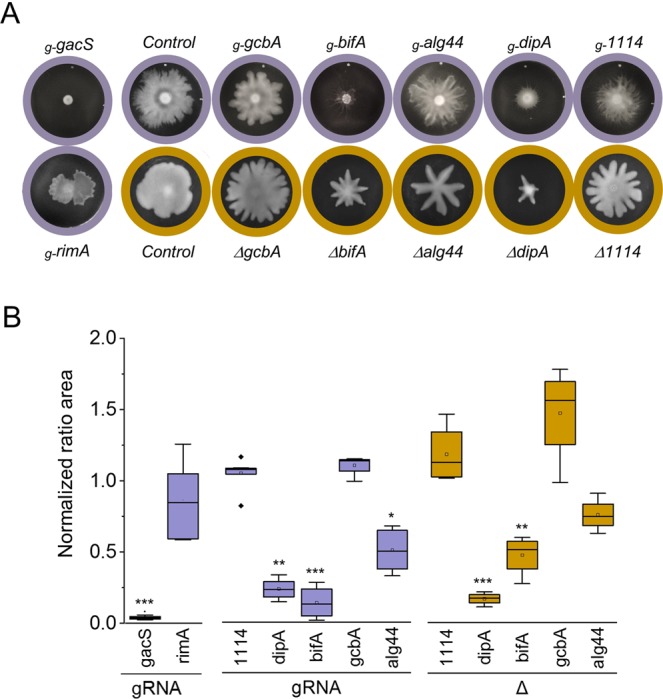
Swarming motility phenotypes in *P*. *fluorescens* SBW25. Swarming phenotypes were compared for CRISPRi-mediated silencing and deletion mutants. CRISPRi silencing used gRNA_NT_ targeting the start of the ORF (denoted *g-gene name*). Target genes include *gacS* (PFLU3777) encoding the kinase sensor protein GacS and several genes encoding c-di-GMP binding proteins such as RimA (PFLU0263, PDE), DipA (PFLU0458, PDE), the alginate co-polymerase Alg44 (PFLU0988), GcbA (PFL0621, DGC) and BifA (PFLU4858, PDE) (see also Supplementary Fig. S6). The control corresponds to the pPFL-gRNA plasmid with no guide RNA inserted. Note that SBW25 cells deleted for the same genes also carried the non-active CRISPRi system (i.e., no gRNA expressed) to treat all the strains under identical conditions. (**A**) Typical swarming morphotypes observed at the surface of soft-agar plates incubated 48 hrs at 25 °C. Plates are circled in purple for CRISPRi silencing (gRNA) and in brown for deletion mutants (Δ). (**B**) Box plot representing the swarm areas of cells with silenced (gRNA) or deleted (Δ) gene relative to the control strain (n ≥ 4). Statistical significance is indicated (T test, *p <  = 0.05 **p <  = 0.01 ***p <  = 0.001).

**Figure 4:**
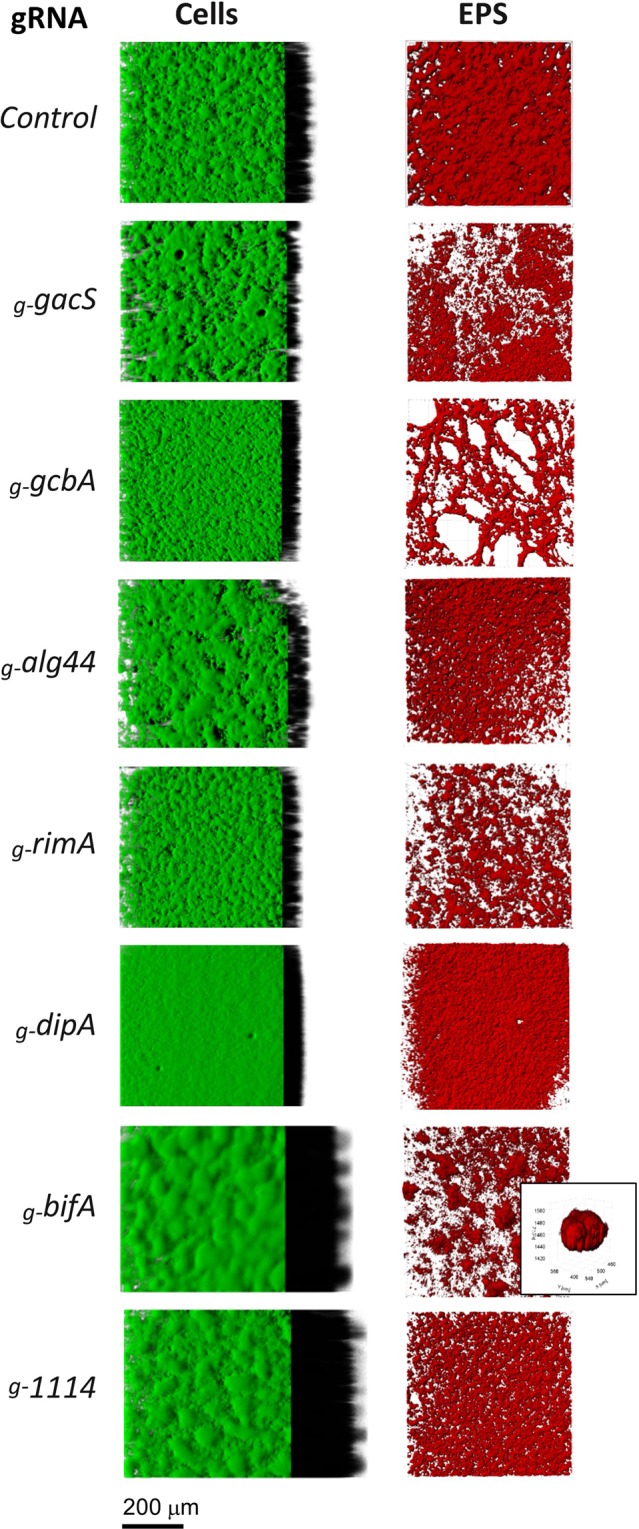
CRISPRi-mediated phenotypes associated with biofilm structure and EPS matrix. *P*. *fluorescens* SBW25 cells expressing the CRISPRi system targeting genes *gacS* (PFLU3777, two-component sensor kinase), *rimA* (PFLU0263, PDE), *dipA* (PFLU0458, PDE), *alg44* (PFLU0988, alginase co-polymerase Alg44), *gcbA* (PFL0621, DGC) and *bifA* (PFLU4858, PDE). The control corresponds to the pPFL-gRNA plasmid with no guide RNA inserted. (A) Cells were stained with FM 1–43 and observed by confocal microscopy. 3D architecture of biofilms were reconstructed as described in Methods. Virtual shadow projections were included on the right to show thickness. (B) Biofilms grown in the presence of the Congo Red dye to reveal the structure of EPS. The ESP-stained volumes were rendered with maximum intensity projection. Inset shows a blow-up view of a clump in the EPS matrix after silencing of *bifA*.

**Figure 5:**
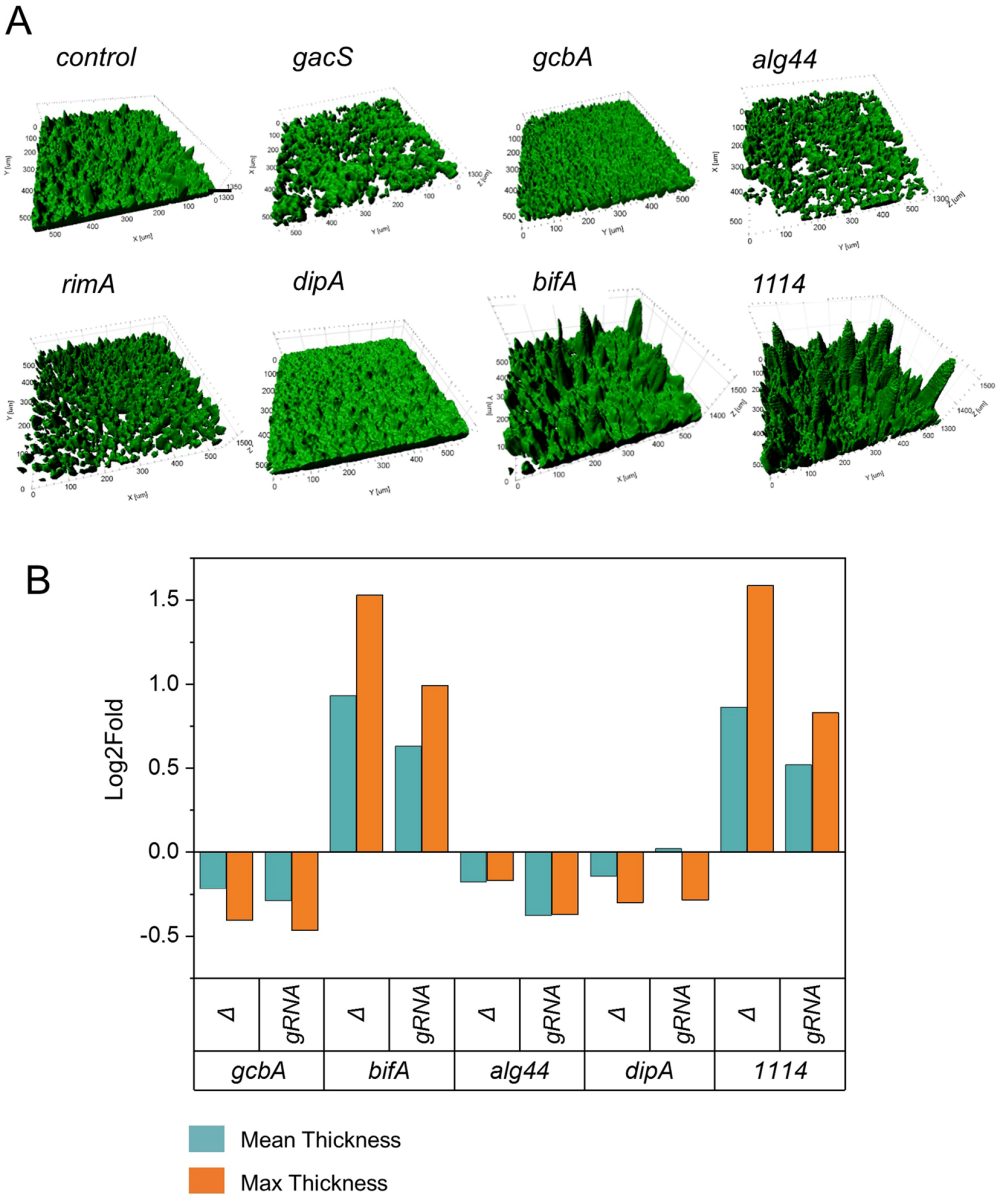
Biofilm phenotypes of genes involved in GacS and c-di-GMP signaling are consistent for CRISPRi silencing and deletion. (**A**) Surface rendering (IMARIS software) of reconstituted 3D confocal volume images of biofilm pellicles displayed in Fig. 4. Targeted genes encode the GacS (PFLU3777) kinase sensor and the c-di-GMP binding proteins RimA (PFLU0263, PDE), DipA (PFLU0458, PDE), Alg44 (PFLU0988) alginate co-polymerase, GcbA (PFL0621, DGC) and BifA (PFLU4858, PDE). The control corresponds to the pPFL-gRNA plasmid with no guide RNA inserted. (**B**) Comparison of changes in biofilm thickness caused by CRISPRi silencing and gene deletion.

CRISPRi system has been shown to confer a rapid gene silencing that is stable over time^54,66^. As numerous *P*. *fluorescens* phenotypes related to mobility and biofilm are typically measured after 48 hours, we investigated CRISPRi-mediated silencing of the pleiotropic *gacS* (PFLU3777) gene. Upon induction of *dCas9* expression, SBW25 cells expressing the *gacS*_*NT*_ guide were totally impaired for swarming motility after 48 hours (Fig. 3), in keeping with the tight control of motility by *gacS* in *Pseudomonas*^17,24,25,67,68^. Expression of the *gacS*_*T*_ guide also led to a substantial defect in swarming (Supplementary Fig. S6). Silencing of *gacS* produced strong defects in biofilm formation relative to the control strain with no gRNA (Figs 4, 5). The biofilm pellicles formed at the air-liquid interface were imaged by confocal microscopy after staining of the cells with the membrane-specific dye FM1-43 biofilm tracer (green) or staining of the exopolysaccharides with the Congo red dye (red). 3D reconstruction of biofilms revealed that *gacS* silencing produced a thinner and less cohesive pellicle with lower cell biomass compared to control (Figs 4, 5A). Likewise, the exopolysaccharide matrix exhibited irregular density and discontinuities, indicating impaired biofilm formation and altered physical properties of the pellicle. Thus, CRISPRi-mediated silencing of *gacS* fully reproduces the phenotypic defects in surface motility and biofilm formation that are hallmarks of *gacS* mutants in *Pseudomonas*^17,18,20,24,25^. Interestingly, the *gacS* knockdown strain appeared more tolerant to H_2_O_2_ exposure than the control strain (Supplementary Fig. S7). This is in contrast with previous observations that growth of SBW25 *gacS*::Tn5 is inhibited in the presence of H_2_O_2_^25^ and the difference may be due to our use of a short and acute H_2_O_2_ treatment. We conclude that CRISPRi silencing enables the reliable study of plate-based phenotypes that take 48 hours to develop. It is therefore a robust approach to investigate gene-phenotype relationships in *P*. *fluorescens*. Silencing of *gacS* was used as internal control in all our CRISPRi assays for swarming and biofilm formation.

### CRISPRi silencing of genes involved in c-di-GMP signaling likely affects other genes in operons

CRISPRi silencing is known to be polar and affect gene expression at the operon level^56,69^. Thus, we evaluated operonic structures for six genes in SBW25 that encode c-di-GMP binding proteins (Supplementary Figure S3). These genes include PFLU0988 (*alg44*), a homolog of *P*. *aeruginosa alg44* encoding an alginate co-polymerase^70^; PFLU0621 (*gcbA*) encoding the DGC-promoting biofilm enzyme GcbA^33^; PFLU0458 and PFLU4858 which are homologs of *dipA* and *bifA*, respectively, and encode enzymes with characterized PDE activity in *P*. *aeruginosa and P*. *putida*^35,37–39^; PFLU0263 (rimA) encoding the single EAL domain protein RimA with characterized PDE activity in SBW25^41^. These genes are known to act at various stages of the biofilm formation in *P*. *aeruginosa*^28^. In addition, we investigated PFLU1114, a gene of unknown function, encoding a putative PDE (Pseudomonas Ortholog Group POG020457) recently shown not to affect motility^71^. Regarding operons, we examined our previous SBW25 gene expression profiles obtained under growth in conditions unrelated to biofilm formation^72^ (Supplementary Fig. S8). These profiles revealed that *rimA*, *dipA* and PFLU1114 are the first genes of their operons. Of note, downregulation of *rimA* is known to affect expression of the downstream genes *rimB* (PFLU0262) and *rimK* (PFLU0261)^41^. The *bifA* gene, although described as a single transcription unit^73^ is part of an operon and likely coexpressed with the downstream gene PFLU4859. The *gbcA* gene appears as a single cistron under the growth conditions examined. However, its genomic arrangement suggest it could potentially be co-expressed with the downstream PFLU0620 gene under other conditions (Supplementary Fig. S8B). Finally, *alg44* is the third gene of a 12-gene cluster that compose the *algD* operon. The *algD* operon is known to be regulated by the alternative stress sigma factor σ22 in Pseudomonas^74,75^, in keeping with our observation that it is repressed under non-biofilm growth condition (Supplementary Fig. S8B). Based on these observations, our six genes of interest are part of operons and their silencing is likely to affect the transcription of neighbor genes. For each gene, we designed a gRNA_NT_ targeting the start of the ORF (Supplementary Table S1) and constructed a deletion mutant (see Methods), except for *gacS* and *rimA* which have been extensively studied. Then, we measured the effects of gene silencing and gene deletion on phenotypes associated with swarming motility and biofilm formation (see below).

### Effects of CRISPRi-mediated silencing on swarming motility

To compare swarming areas under identical conditions for strains with CRISPRi silencing and deletions, all the deletion mutant strains were transformed with both pPFL plasmids expressing *dCas9* and control (no-guide) gRNA. Cells were grown on 0.4% agar contain plates in the presence of kanamycin (50 μg/ml), gentamycin (10 μg/ml) and aTc (100 ng/ml), and swarming areas were measured after 48 h at 25°C. CRISPRi silencing of *rimA*, *bifA* and *dipA* drastically reduced swarming ability whereas silencing of *gcbA*, *alg44* and PFLU1114 did not substantially affect swarming (Fig. 3A). The swarming phenotypes obtained upon silencing with gRNA_T_ were consistent but of reduced amplitude relative to those obtained with gRNA_NT_ in line with less efficient silencing when targeting template DNA strand (Supplementary Fig. S6). We found that swarming phenotypes observed with CRIPSRi were in good agreement with those from deletion mutants (Fig. 3A), albeit with a larger standard deviation for *Δ*1114 and *ΔgcbA* (Fig. 3B). Together these results highlighted the relevance of CRISPRi-based approach to study loss-of-function motility phenotypes.

### Silencing of genes involved in c-di-GMP signaling provide insight in biofilm formation and structure

To assess CRISPRi-mediated gene silencing on biofilm formation, bacterial cells and EPS present in air-liquid pellicles were dyed separately and imaged by confocal microscopy (see Methods). Biofilms formed by RimABK-depleted cells were thinner, and flakier than the control with an irregular EPS matrix structure (Fig. 4), in agreement with phenotypes previously observed in a SBW25 *ΔPFLU0263* strain^41^. Biofilms formed by DipA-depleted cells appeared very flat and dense with a homogenous repartition of EPS (Fig. 4). In contrast, the depletion of BifA gave rise to a substantial increase of biofilm thickness and formation of clumps in the EPS matrix. The largest increase in biofilm mass was observed upon depletion of PFLU1114, which did not alter the texture of the EPS matrix but produced a particularly thick biofilm (Fig. 4). Depletion of the alginate co-polymerase Alg44 resulted in a thinner biofilm with more dispersed EPS matrix than the control, in keeping with its role in synthesis of the exopolysaccharide alginate^76^. Finally, the depletion of GcbA only slightly impaired biofilm cell thickness but produced an altered EPS matrix (Fig. 4).

The reconstructed 3D volumes of biofilms were quantitatively analyzed for thickness and roughness (Fig. 5A, Supplementary Table S4). Phenotypes mediated by CRISPRi were highly consistent with those from deletion mutants (Fig. 5B, Supplementary Table S4). In both deletion and depleted conditions, a slight but statistically significant decrease in biofilm thickness was observed for *gcbA* and *alg44* and no significant change was found for *dipA*. A more prevalent thickness was also observed in deleted *vs* depleted conditions for *bifA* and PFLU1114 with a statistically significant increase in mean (∼1.5 fold) and max thickness (∼2 to 3 fold), and roughness (∼2.5 fold) relative to the control. This difference could reflect partial gene silencing in our experimental conditions. In contrast, the roughness was slightly less pronounced in deleted compared to depleted strains.

**Figure 6:**
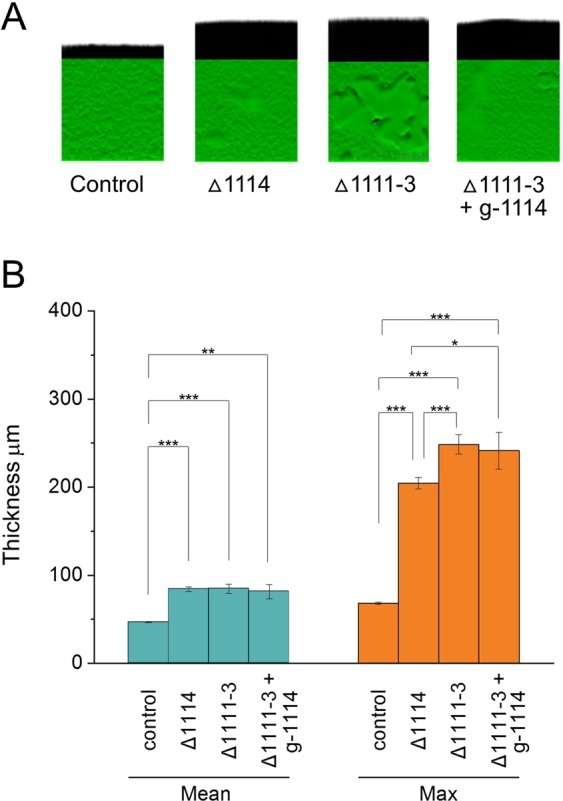
Role of the PFLU1114 operon in biofilm mass and structure. Quantification of the 3D reconstructed volumes in air-liquid biofilm pellicles (see Fig. 4) were performed using IMARIS x64 9.0.2 XTension software package. (**A**) Pseudo-3-D images with shadow projection. (**B**) Histograms displaying the distribution of data obtained from observation of biofilm pellicles (n ≥ 6) from two independent experiments. Pairwise comparisons were performed using the Tukey Method (*p <= 0.05 **p <= 0.01 ***p <= 0.001).

We investigated whether the increased biofilm cell mass, thickness and roughness observed upon PFLU1114 silencing was due to PFLU1114 alone or to a polar effect on the downstream PFLU1111-1113 genes. The deletion of PFLU1111-1113 genes (*Δ1111-3*) exhibited similar effect on biofilm than the deletion of PFLU1114 (*Δ*1114) alone (Fig. 6A). Furthermore, the CRISPRi silencing of PFLU1114 in the *Δ1111-3* strain did not change significantly mean thickness compared to the deletion mutants (Fig. 6B). A slight but statistically significant increase in max thickness was observed in the *Δ1111-3* strain and it was not further affected by depletion of PLU1114. These results indicate that proteins encoded by the whole PFLU1114 operon act together to regulate biofilm mass and thickness.

## Discussion

We adapted the CRISPRi system to work in three genetically and physiologically diverse *P*. *fluorescens* strain isolates: SBW25, WH6 and Pf0-1^77^. CRISPRi effectively inhibited the expression of a constitutively expressed fluorescent reporter in all three strains, suggesting that our system can be utilized across the *P*. *fluorescens* group. The amplitude of the inhibition depended on the DNA site and strand targeted by the gRNA and the concentration of aTc inducer, demonstrating that these features can be used to modulate the level of inhibition. We elected to design gRNAs that bind to the start of the ORFs and are complementary to the protein coding strand because these features yielded a substantial inhibition of gene transcription (30-fold) and protein expression (8.7-fold in SBW25) with a homogeneous distribution of fluorescence intensity in the cell population (Fig. 1). These findings indicate that the induction of dCas9 blocks transcription elongation resulting in efficient gene silencing.

Silencing by CRISPRi was used in SBW25 to investigate genes involved in cell division and cell morphology for which phenotypes are observed after 3–18 hours of growth, and genes involved in the regulation of motility and biofilm formation for which phenotypes are scored after 48 hours on plate and liquid culture assays. We found that CRISPRi-mediated silencing of *ftsZ* and *mreB* genes caused characteristic cell morphology defects in the whole cell population. Silencing of genes involved in signaling pathways that control swarming motility and biofilm formation produced phenotypes that are consistent with phenotypes of gene inactivation mutants observed in this study. Our analysis also revealed novel phenotypes related to the EPS matrix for previously studied genes (e.g., *gcbA*, *bifA*), and allowed the discovery of a key role of the PFLU1111-4 operon in inhibiting biofilm formation in SBW25. Recently, a CRISPRi system based on *S*. *pasteurianus* dCas9 was successfully tested in *P*. *aeruginosa* and in other *Pseudomonas* but the described leakiness of the promoter controlling *SpadCas9* expression represented a limitation for phenotype analysis^78^. Here, our CRISPRi system is a robust and reliable approach to study complex phenotypes related to *P*. *fluorescens* life styles and behaviors, potentially opening the way to more systematic studies in this diverse bacterial group. However, because of polar effects caused by CRISPRi, an important limitation is that a phenotype can only be attributed to an operon^69^. Identification of the particular gene or subset of genes causing the phenotype will require additional investigations using other approaches (e.g., gene deletion and complementation in *trans*).

In *P*. *fluorescens* SBW25, we found a strong effect of GacS depletion on swarming, in agreement with previous observations that swarming motility was reduced in a *gacS::Tn5* mutant^25^. In SBW25, GacS also regulates genes involved in oxidative stress response, and the *gacS::Tn5* mutant has a reduced capacity to survive chronic exposure to sub-lethal doses of H_2_O_2_^25^, which is correlated with a decreased expression of *katE* encoding a catalase. Upon silencing of *gacS* with CRISPRi, we did not observe such enhanced H_2_O_2_ sensitivity but rather a slight and reproducible increase in H_2_O_2_ tolerance. Interestingly, a strong upregulation (300-fold) of the superoxide dismutase SodA was also observed in the *gacS::Tn5* mutant^25^, suggesting that the higher tolerance we observe upon GacS depletion may result from the short and acute exposure to H_2_O_2_ we applied versus the chronic, low-dose exposure to H_2_O_2_ applied in the previous study^25^.

Our analysis of genes from the c-di-GMP regulatory network revealed that CRISPRi silencing can be used for detailed investigation of biofilm phenotypes and gene function. Targeting of the *rim* operon did not significantly impair swarming motility but strongly affected biofilm formation, in keeping with previous results that swarming in SBW25 is slightly affected by *ΔrimK* deletion while not affected at all by *ΔrimA* and *ΔrimB* deletions^41^. We found that the downregulation of the DGC *gcbA* affects biofilm thickness and structure as well as EPS production. In *Pseudomonas*, GcbA plays a key role in transition to irreversible attachment to surface, a process linked to EPS production^33^. CRISPRi silencing of *gcbA* also resulted in a decrease of cell mass and altered structure of the EPS in the biofilm similar to *ΔgcbA*, suggesting an additional role of GcbA in the regulation of EPS matrix formation in SBW25. This is in keeping with phenotypes observed in *P*. *fluorescens* Pf0-1 *ΔgcbA* and *P*. *aeruginosa gcbA::Tn*5 mutants^36,79^. The alginate biosynthesis *algD* operon, containing *alg44* is expressed under specific physiological conditions. In *P*. *putida*, this operon is upregulated under water-limited condition^80^, while in *P*. *aeruginosa*, it is expressed under iron limitation^81^. The defects in SBW25 biofilm formation observed upon depletion or deletion of *alg44* indicate that the *algD* operon is upregulated under our condition for biofilm formation. Since *alg44* is the third gene of the *algD* operon, the phenotypes we observed could be explained by polar effects of CRISPRi silencing that affect the expression of downstream and upstream genes, including *algD*. Such phenomenon has been previously reported in *B*. *subtilis*, illustrating the polar effects of CRISPRi at the level of operons^56,69^.

Major changes in biofilm architecture were also observed after depletion of PDEs. The silencing of *bifA* and *dipA* produced extreme but opposite phenotypes related to biofilm mass, thickness and structure. These observations support the notion that biofilm formation is not directly promoted by a higher concentration of c-di-GMP in the cell but is regulated by discrete and interconnected pathways that respond to local concentrations of c-di-GMP^26^. The DipA protein plays a crucial role in biofilm formation and dispersion in *P*. *aeruginosa* as a *ΔdipA* mutant exhibits reduced swarming motility, increased EPS production and reduced cell dispersal^35^. Our observations are in line with these results and further reveal the denser and smoother biofilms formed by DipA-depleted cells, potentially accounting for the reduced cell dispersal. In contrast, we found that the depletion of BifA leads to a large increase in biofilm thickness and 3-dimensional architecture with the formation of mushroom cap-shaped clumps in the ESP matrix. This corroborates the phenotypes of a *ΔbifA* mutant in *P*. *aeruginosa*, which exhibits a hyper biofilm phenotype as well as a loss of swarming ability^38,82^. Our findings also reveal a role of BifA in controlling spatial organization and structure of EPS in SBW25. Finally, we discovered that the depletion of PFLU1114 triggers the formation of biofilms that are remarkably thick and highly structured. Because the swarming motility and the synthesis of EPS were not affected by PFLU1114 depletion, we hypothesize that PFLU1114 acts after the attachment stage to limit the cell density in the maturing biofilm. By combining gene deletion and CRISPRi silencing, we also established that this role in biofilm control involves the whole PFLU1114 operon. In addition to GGDEF and EAL c-di-GMP-related domains, the PFLU1114 protein possesses a CheY-like REC module present in signal transduction response regulators (Supplementary Fig. S3). The operon also encodes a two component histidine kinase system (PFLU1112-1111)^46^. Future investigations will be necessary to understand how functions of the PFLU1114-1111 proteins integrate with the c-di-GMP signaling pathway to regulate biofilm formation in *P*. *fluorescens*.

CRISPRi silencing is an appealing approach for future systematic interrogation of gene networks in *P*. *fluorescens*. Such approaches have been successfully applied in other bacteria to identify essential genes^56,83^. Our CRISPRi system could be applied to investigate systematically all the genes involved in signaling pathways that regulate bacterial life styles, including biofilm formation. A single strain can be transformed with a combination of two plasmids, producing strain derivatives with identical genetic backgrounds. These cells can be propagated without inducer, potentially limiting the selective pressure caused by CRISPRi activity and reducing the probability for spontaneous accumulation of adaptive mutations that restore cell fitness, as observed in cells carrying gene deletions^84–86^.

## Methods

### Microbial strains and media

*P*. *fluorescens* strains SBW25, and WH6 and Pf0-1 genetically labelled by the mNeongreen (mNG) fluorescent protein expressed from a constitutive promoter (Pc) were described in a previous study^11^. Plasmids were constructed and propagated in *E*. *coli* DH5α (Biolabs) prior to transformation in *Pseudomonas*. Bacterial cultures and phenotype assays (e.g., swarming motility) were performed in LB medium (liquid or agar-containing) and in M9 medium supplemented with glucose 0.4% as carbon source. When appropriate, kanamycin (50 μg/ml) and gentamycin (10 μg/ml) were added to select for plasmid maintenance. Anhydrotetracycline (aTc) was used as inducer of the P_tetA_/TetR promoter/repressor system at the indicated concentrations. Cells were grown with shaking at 37 °C for *E*. *coli* and 28 °C for *P*. *fluorescens*. Swarming and biofilm assays with *P*. *fluorescens* were performed in a humidity-controlled growth chamber at 25 °C under 70% humidity.

### Construction of CRISPRi vectors

pPFL-dCas9 was constructed by insertion of a PCR-amplified DNA fragment carrying the minimal replicon sequence of the *P*. *fluorescens* plasmid pVS1^87^ into the BsrGI/StuI restriction sites of plasmid pAN-PTet-dCas9 vector^57^. The resulting plasmid pPFL-dCas9 can be stably propagated in *P*. *fluorescens* and maintained by applying kanamycin selection. In pPFL-dCas9, the gene encoding dCas9 is placed under a Tetracyclin-inducible P_tetA_ promoter controlled by the *tetR* repressor gene (Fig. S1). The pPFL-gRNA was built by insertion of a PCR-amplified DNA fragment carrying the constitutive J23119 promoter, dCas9 handle and *S*. *pyogenes* terminator cassette from plasmid pgRNA-bacteria^55,88^ into the EcoRI/PpuMI restriction site of plasmid pSEVA643^89^. The resulting pPFL-sgRNA plasmid is compatible with pPFL-Cas9 in *P*. *fluorescens*, can be maintained with gentamycin selection, and serves as backbone for the synthesis of gRNAs (Fig. S1). The gRNA sequences were copied either from the template or to the non-template DNA strands of the targeted genes (Fig. S2). The ‘CasFinder’ software package (https://omictools.com/casfinder-tool) was used to design gRNAs with minimal potential for off-target effects^90^. The gRNA sequences (Table S1) were synthetized as DNA gBlocks (Integrated DNA technologies, https://www.idtdna.com) and cloned into the EcoRI/SpeI restriction sites of pPFL-gRNA. Plasmid constructs were transformed in *E*. *coli*, purified and transferred in *P*. *fluorescens* strains SBW25, WH6 and Pf0-1, using standard electroporation techniques^91^.

### Construction of deletion mutant strains

Gene deletions were carried out using a λ-Red recombineering system developed in *P*. *fluorescens* SBW25^11^. Gene replacement by a tetracycline resistance selection marker was performed as described elsewhere^11^. Briefly, linear DNA fragments containing 500 pb of flanking regions 3’ and 5’ of the targeted genes and the tetracycline resistant cassette were generated by PCR and assembly cloning, The resulting 2 kb fragments were then transformed into *P*. *fluorescens* SBW25 cells harboring a plasmid expressing the RecET recombinases under the control of an arabinose-inducible promoter.

Transformants were selected for tetracycline resistance (10 μg/ml). Chromosomal constructs were verified by PCR and strains were further cured from recombineering plasmid prior to transformation by plasmids carrying the CRISPRi system. The oligonucleotides used to generate the fragments used in gene assembly are indicated in Supplementary Table S3.

All the deleted strains were further transformed by the pPFL-dCas9 and negative control (non-targeting) pPF-sgRNA plasmids allowing apply identical selective pressure than during the CRISPRi- mediated silencing assays.

### Analysis of CRISPRi-mediated phenotypes

#### Fluorescence signal by flow cytometry

*P*. *fluorescens* strains expressing the mNeonGreen fluorescent protein and carrying plasmids pPFL-dCa9 and pPFL-gRNA derivatives were grown at 28 °C in LB medium supplemented with kanamycin (50 μg/ml) and gentamycin (10 μg/ml). Overnight cultures were diluted to OD_600_ 0.1 in fresh LB medium supplemented with the same antibiotics and with or without the inducer anhydrotetracyclin (aTc) 100 ng/ml and cultures were grown at 28 °C under agitation for 7 hours. Culture samples (10 µl) were taken at 0, 1, 3, 5 and 7 hours, diluted in PBS and subjected to flow cytometry (CytoFlex S, Beckman) to quantify green fluorescence. At least 10^4^ particles were counted for each sample. Computerized gating in forward scatter (FSC) and side scatter (SSC) was used to eliminate cell debris. Histograms were generated using the Kaluza 2.0 software (https://www.beckman.com/coulter-flow-cytometers/software/kaluza).

#### Morphological defects by epifluorescence microscopy

Cells carrying pPFL-dCas9 and pPFL-gRNA derivatives were grown at 28 °C in LB supplemented with kanamycin (50 μg/ml) and gentamycin (10 μg/ml). Overnight cultures were diluted in fresh LB supplemented with the same antibiotics to OD_600_ 0.1 with or without aTc concentrations ranging from 100–500 ng/ml. Culture samples were taken at various times and mixed with the membrane dye FM4-64 (Invitrogen) prior to immobilization on glass slides padded with agarose 1.3%. Images were acquired using a fluorescence microscope (Nikon Eclipse Ti) equipped with a Plan Apo λ 100 × /1,45 NA oil (WD = 0.13 mm) and filter set compatible with red (excitation wave length 555 nm, emission 630 nm). After imaging, the fluorescence signal was false colored in red using the NIS-element software to highlight bacterial membranes.

#### Bacterial motility phenotypes

The *P*. *fluorescens* SBW25 derivatives containing pPFL-dCas9 and pPFL-gRNA derivatives were first streaked on LB agar containing kanamycin (50μg/ml) and gentamycin (10 μg/ml) and grown at 28 °C. A single colony was used to inoculate in LB medium containing kanamycin and gentamycin and allow to grow at 28 °C with shaking at 220 rpm. Overnight cultures from all strains were adjusted to OD_600_ = 1 and a 1.5 μL drop was deposited at the center of the swarming plate (60 mm × 15 mm) containing semi-solid LB-agar (0.4%), antibiotics and aTc at 100 ng/ml. Plates were incubated at 25 °C for 48 hours before imaging. Swarming surface areas were assessed by imageJ (https://imagej.nih.gov/ij/) and normalized compared to the swarm area of the control strains containing pPFL-dCas9 and a pPFL-gRNA derivative with no guide RNA. Box plots of the distributions of numerical data obtained from ImageJ were displayed by Origin(Pro), Version Number (e.g. “Version 2019b”). OriginLab Corporation, Northampton, MA, USA).

#### Survival after exposure to acute oxidative stress

Strains were inoculated in LB supplemented with kanamycin 50μg/ml and gentamycin 10 μg/ml and grown overnight at 28 °C with shaking at 220 rpm. Cultures were then diluted to OD_600_ 0.02 in fresh media and allowed to grow up to OD_600_ 0.2–0.3 prior to addition of aTc 100 ng/ml and additional cultivation for 4 hours at 28 °C. All cultures were then adjusted to OD_600_ = 1 prior to addition of H_2_O_2_ at 2.5, 5 and10 mM final concentrations and incubated for 30 min at 28 °C. Cells were then collected, serially diluted in fresh LB and plated on selective LB agar plates. Bacterial viability was measured by counting the colony forming units after 24 h at 28 °C. Results were analyzed using Student’s t-test. P-value < 0.05 were considered statistically significant.

#### Biofilm formation

Cells carrying pPFL-dCas9 and pPFL-gRNA derivatives were grown at 28 °C in LB supplemented with kanamycin (50 μg/ml) and gentamycin (10 μg/ml). Overnight cultures were diluted in fresh M9-glucose supplemented with the same antibiotics to OD_600_ 0.1 and grown at 28 °C for 5 hours. Cultures were adjusted to identical OD_600_ and inoculated to OD_600_ = 0.1 in triplicates in 12 wells culture plates containing fresh selective M9-glucose media supplemented with aTc 100 ng/ml. Two additional wells were inoculated in the presence of Congo Red (CR). Plates were incubated at 25 °C and 70% humidity in a growth chamber for 48 h. Biofilm pellicles were carefully brought up to the top of the wells by slowly adding M9 media along the side of the well and then peeled off on a 25 mm diameter cover glass slide. The cover slides with intact biofilm pellicles were then mounted on an Attofluor™ Cell Chamber, covered with 1 ml of PBS or transparent minimal media and stained with the FilmTracer FM 1–43 Green Biofilm dye (Molecular Probe) for 30 min prior to observation by confocal microscopy. Pellicles dyed with Congo red were mounted similarly and observed directly.

Stained biofilms were observed using a spinning disk confocal microscope (Nikon Eclipse Ti-E coupled with CREST X-Light^TM^ confocal imager; objectives Nikon CFI Plan Fluor 10 × , DIC, 10 × /0.3 NA (WD = 16 mm)). Excitation was performed at 470 nm and emission recorded at 505 nm (green). Congo Red stained cells were observed using excitation and emission wave lengths of 555 and 600 nm, respectively. Images were processed using IMARIS software (Bitplane, South Windsor, CT, United States). Biofilm images were quantified using the surface function in IMARIS (XTension biofilm). Means and maxima for surface thickness, roughness and surface substratum were determined from at least 3 independent pellicles and 2 measurements per pellicle Box plots of the distributions of numerical data obtained from Imaris were displayed by Origin(Pro), Version Number (e.g. “Version 2019b”). OriginLab Corporation, Northampton, MA, USA).

## Supplementary information

Supplementary information

## Data Availability

All data generated or analyzed during this study are included in this published article (and its Supplementary Information files).
