# Supplementary material for: CRISPR interference to interrogate genes that control biofilm formation in Pseudomonas fluorescens
Source: Sci Rep. 2019 Nov 4;9:15954. doi: 10.1038/s41598-019-52400-5 (PMC6828691; doi:10.1038/s41598-019-52400-5)
Supplement: Supplementary file 1 — Supplementary information [file 41598_2019_52400_MOESM1_ESM.docx]

**CRISPR interference to interrogate genes that control biofilm formation in *Pseudomonas fluorescens***

# Marie-Francoise Noirot-Gros^1^*, Sara Forrester^1^, Grace Malato^1^, Peter Larsen^1^ and Philippe Noirot^1^

1Biosciences Division, Argonne National Laboratory, Lemont, IL, United States.

* Corresponding author: [mnoirot@anl.gov](mailto:mnoirot@anl.gov)

Supplementary Information

**Supplementary Figures:**

**Supplementary Figure S1: CRISPR Interference System in *P. fluorescens*.**

**Supplementary Figure S2:** Determining optimal conditions for CRISPRi silencing in various *P. fluorescens* strains.

**Supplementary Figure S3:** Genomic organization of the genes targeted for silencing and annotation of protein functional domains**.**

**Supplementary Figure S4.** CRISPRi silencing of *ftsZ* and *mreB* using non-template strand gRNA.

**Supplementary Figure S5.** CRISPRi silencing of *ftsZ* and *mreB* using template strand gRNA.

**Supplementary Figure S6.** Effects of gRNA strand selection on swarming phenotypes.

**Supplementary Figure S7.** CRISPRi silencing of *gacS* increases cell survival to acute oxidative stress.

**Supplementary Figure S8A,B.** Transcriptional landscapes of c-di-GMP genes.

**Supplementary Tables:**

**Supplementary Table S1.** List of gBlocks gene fragments for gRNA designs.

**Supplementary Table S2.** Quantification of CRISPRi-mediated mNG gene silencing in *P. fluorescens*.

**Supplementary Table S3.** List of oligonucleotides for generation of gene deletions

**Supplementary Table S4.** Statistical comparison of CRISPRi-gene targeted knockdowns and gene knockouts in biofilm phenotypes

A

**
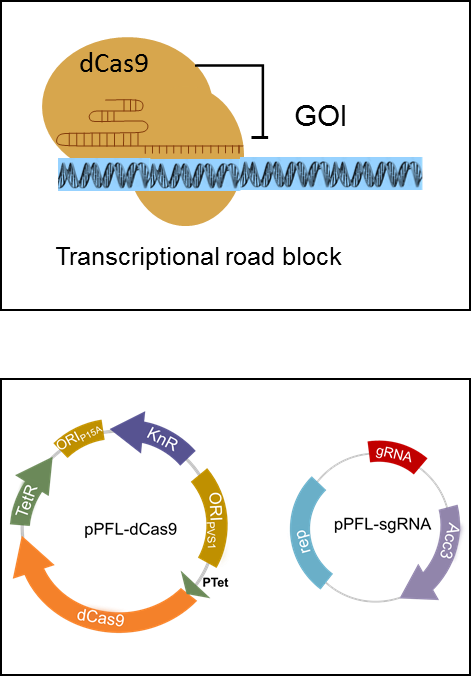
**

B

**Figure S1: CRISPR Interference System in *P. fluorescens***

**(A)** The catalytically inactive dCas9 protein binds the sgRNA chimera that will guide the protein-RNA complex to bind to a targeted gene and act as a transcriptional road block.

(B) The plasmid maps of the gRNA and dCas9 expression vectors. The pPFL-sgRNA plasmid express the gRNA from a constitutive promoter (pJ23119) with a strong terminator, rrnB, to ensure efficient termination, a gentamycin-selectable marker (Acc3) and a ColE1 replication origin, which is active in *E. coli* and *P. fluorescens*. The pPFL-dCas9 plasmid contains an aTc-inducible promoter pLtetO-1 under control of the TetR repressor, a strong ribosomal binding site (RBS), a kanamycin-resistance marker (KnR), and two ORI regions composed of the P15A replication origin, functional in *E. coli* and the pVS1 replication origin functional in *P. fluorescens*.

**
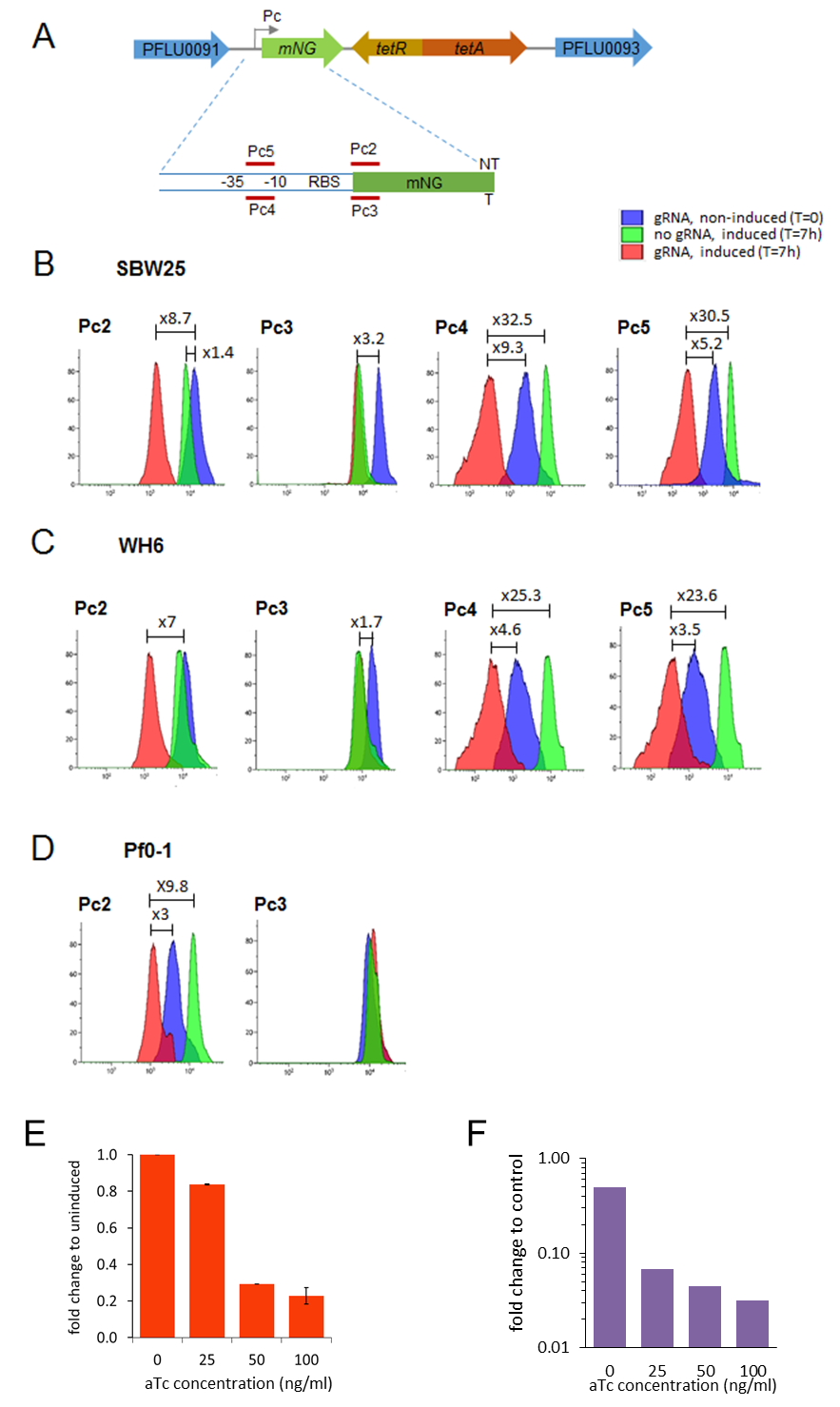
**

**Figure S2**

**Figure S2**: **Determining optimal conditions for CRISPRi silencing in various P. *fluorescens* strains**

The *mNG* gene encoding the mNeonGreen fluorescent protein has been inserted at similar, presumably neutral, locations in the genomes of SBW25, WH6 and Pf0-1, as previously described ^1^. (**A**) Genetic organization of the SBW25 chromosome surrounding the *Pc-mNG* construct used as a reporter for the activity of our CRISPRi system. A zoom-in section of the Pc region displays the positions targeted by the gRNAs (Pc2-5) relative to the promoter -35 and -10 motifs and the strand they copy. Cultures of cells carrying plasmids pPFL-dCas9 and pPFL-gRNA containing no-guide sequence (as control) or a Pc-guide targeting the mNG gene, were prepared as described in Methods. Expression of dCas9 was induced by addition of aTc to the culture medium (T=0). Cells fluorescence intensities were analyzed by flow cytometry (CytoFlex S, Beckman) after 7 hours a: (**B**) SBW25; (**C**) WH6; and (**D**) Pf0-1. At least 10^4^ particles were measured for each sample. Computerized gating in forward scatter (FSC) and side scatter (SSC) was used to eliminate cell debris. Histograms were generated using the Kaluza 2.0 software. In Pf0-1, peaks became very broad and overlapping when Pc4 and Pc5 gRNAs were used (data not shown). Cells expressing the Pc2 gRNA (NT strand, elongation block) displayed a decrease in fluorescence of 8-, 7- and 10-fold after 7 hours in SBW25, WH6 and Pf0-1, respectively, compared to cells expressing no guide. The silencing of *mNG* expression was much weaker (SBW25, WH6) or not detectable (Pf0-1) in cells expressing Pc3 gRNA (T strand, elongation block). When cells express the Pc4 and Pc5 gRNAs (initiation block), irrespective of T or NT strand, a large decrease in fluorescence was observed in SBW25 and WH6. Thus, repression by initiation-blocking guides in *P. fluorescens* appears to be independent of the targeted DNA strand, in keeping with previous observations in *E. coli*. Importantly, comparison of fluorescence intensities between no gRNA, induced (T=7h) and gRNA, non-induced (T=0) conditions reveals a lower fluorescence when gRNA is present in absence of inducer. This holds true for SBW25 and WH6 strains expressing Pc4 and Pc5, and for Pf0-1 expressing Pc2 gRNA. These findings suggest that dCas9 is expressed as a basal level in our system. Based on these observations, we selected Pc2-like gRNAs (NT strand, elongation block) for our experiments, unless otherwise indicated. Using Pc3-like gRNAs (T strand, elongation block) may provide a way for a moderate down-regulation of gene expression in SBW25 and WH6.

To investigate the dose-dependent downregulation of the mNG gene using the Pc2 gRNA in SBW25, cells were treated with increasing concentrations of aTc for 6 hours and fluorescence intensities were analyzed by flow cytometry. (**E)** Fluorescence intensity decreased with increasing aTc concentration, consistent with dCas9 expression being induced in a dose-dependent manner (results are average of two independent experiments). (**F**) Total RNAs were prepared from cultures treated with various aTc concentrations and quantification of mNG mRNA was performed using qRT-PCR. Abundance of mNG mRNA decreased with increasing aTc concentration, indicating a dose dependent repression of mNG gene expression (up to 30x with 100ng/ml aTc).

1- Noirot-Gros, M. F. *et al.* Dynamics of Aspen Roots Colonization by Pseudomonads Reveals Strain-Specific and Mycorrhizal-Specific Patterns of Biofilm Formation. *Frontiers in microbiology* **9**, 853, doi:10.3389/fmicb.2018.00853 (2018).

**
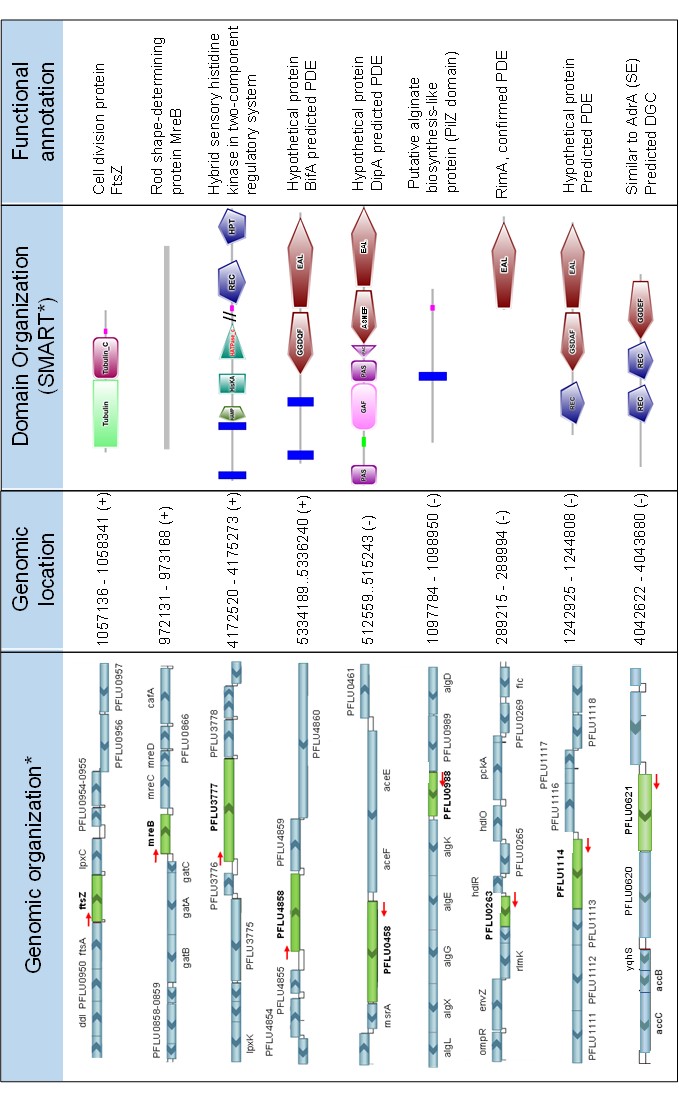
**

**Figure S3: Genomic organization of the genes targeted for silencing and annotation of protein functional domains.**

The organization of the genome around selected genes was extracted from the *P. fluorescens* SBW25 genome in Pseudomonas Genome DB (http://www.pseudomonas.com/). The positions of gRNAs copying the NT strand of targeted genes are indicated by red arrows. Protein Domain Architectures are from SMART (<http://smart.embl-heidelberg.de/smart>).

**Figure S4: CRISPRi silencing of *ftsZ* and *mreB* genes using non-template strand gRNA.**

(A) Cells carry pPFL-dCas9 and pPFL-*ftsZ*_NT_ (gRNA_T_ targeting *ftsZ* gene) , pPFL-*mreB*_NT_ (gRNA_T_ targeting *mreB* gene) or pPFL-control (no gRNA). dCas9 protein was induced by addition of 0.1μg/ml aTc (final concentration). The morphological phenotypes were monitored by epifluorescence microscopy 3 hrs (left panels), 5 hrs (middle panels), and 18 hrs (right panels) after induction. Bacteria were stained by the red fluorescent dye FM4-64 that incorporates into cell membranes. Scale bars correspond to 5μm. (B) Gallery of cells exhibiting morphological defects upon down regulation of *mreB*. White arrow points to a cell burst event.

**Figure S5. CRISPRi silencing of *ftsZ* and *mreB* genes using template strand gRNA.**

Dose responses were monitored in cells carrying pPFL-dCas9 and pPFL-*ftsZ*_T_  (gRNA_T_ targeting *ftsZ* gene). dCas9 was induced with increasing amounts of aTc: (a), 0; (b), 0.02; (c), 0.05; (d), 0.1; (e), 0.2; (f), 0.5 μg/ml; and (g) pPFL-control (no gRNA) strain treated with 0.5 μg/ml aTc. The filamentation phenotype was monitored by epifluorescence microscopy 7 hours after induction. Bacterial membranes were stained with FM4-64. Scale bars correspond to 2μm. (**B**) Induction of the filamentation phenotype over time. Cells expressing the *ftsZ_T_* guide were either not induced (a, b and c) or induced with aT 0.5μg/ml (d, e and f) for dCas9 expression. Cells were stained with FM4-64 and observed by epifluorescence microscopy after 3 hours (a, d), 7 hours (b, e) and overnight (c, f) post induction. Scale bars correspond to 2μm. (**C**) Cells expressing the *mreB*_T_ guide (targeting *mreB* gene at template strand) were induced with 0.5 μg/ml aT and morphological phenotypes were observed after ON growth (18 hrs).

**
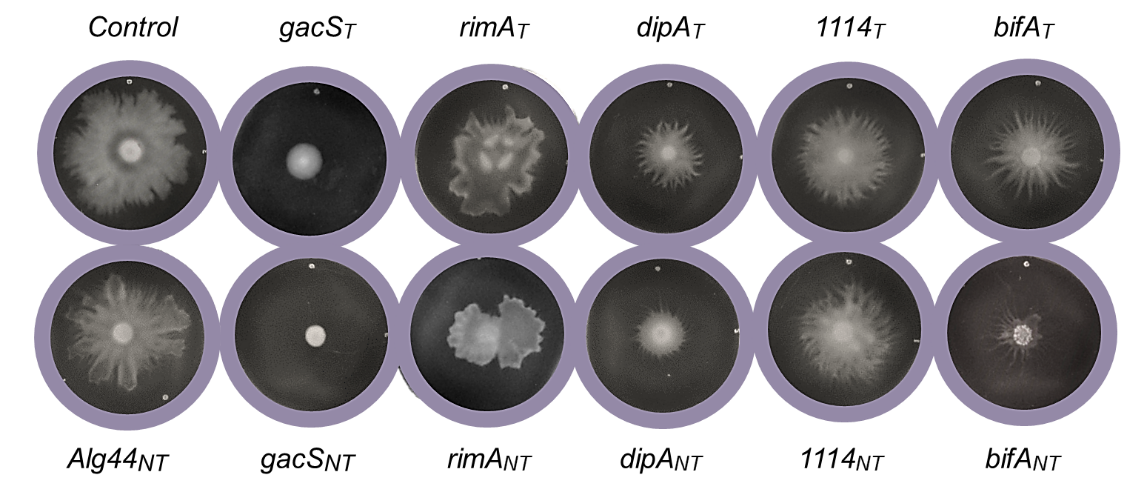
**

**Figure S6. Effects of gRNA strand selection on swarming phenotypes.**

*P. fluorescens* strains harboring pPFL-dCas9 and various pPFL-gRNA expressing either gRNA_NT_ or gRNA_T_ for each targeted gene. In experiments involving gRNA_NT_ and gRNA_T_, dCas9 was induced by 0.1μg/ml and 0.5 μg/ml aTc (final concentrations), respectively. gRNAs target the start of the coding regions of *gacS* (PFLU3777) as well as of genes encoding c-di-GMP binding proteins, *rimA* (PFLU0263), *dipA* (PFLU0458), *alg44* (PFLU0988), *bifA* (PFLU4858) and PFLU1114 (see also Fig. 6). Control corresponds to the pPFL-gRNA plasmid with no guide RNA inserted.

gRNA

H_2_O_2_ (mM)

**Figure S7: CRISPRi silencing of *gacS* increases cell survival to acute oxidative stress**

*P. fluorescens* strain harboring pPFL-dCas9 and expressing the *gacS_NT_* guide to downregulate the two-component kinase gene *gacS* (PFLU3777). Control (-) corresponds to the pFL-gRNA plasmid with no gRNA inserted. Expression of dCas9 was induced 4h prior to exposure of cells to H_2_O_2_ for 30 min. Treated cultures were then serially diluted in LB. (A) Cells were exposed to H_2_O_2_ 10 mM. Serial dilutions were then spotted onto LB agar plates containing kanamycin and gentamycin and incubated for 24 hrs at 28°C. (B) Dilutions were plated on the same medium for colony forming unit (cfu) counts. Cell survival is the number of cfu/ml after 24hrs at 28°C. Experiments were repeated three times independently for each strain and each H_2_O_2_ treatment. Error bars are standard deviation of the means. * P> 0.05, ** P >0.005, *** P>0.0005.


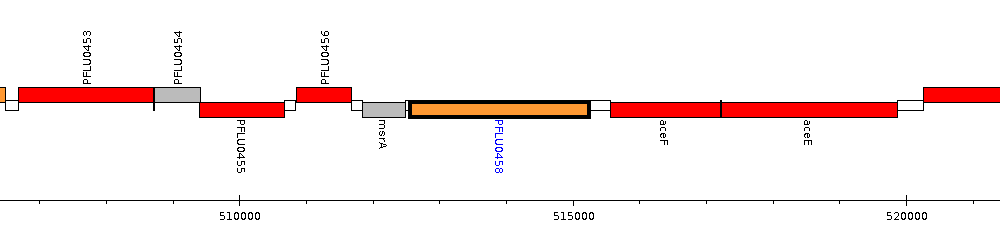

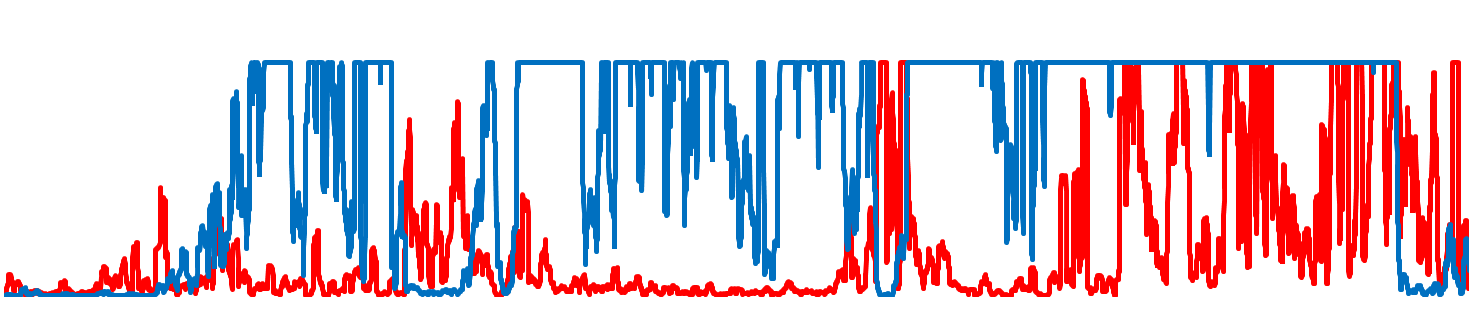

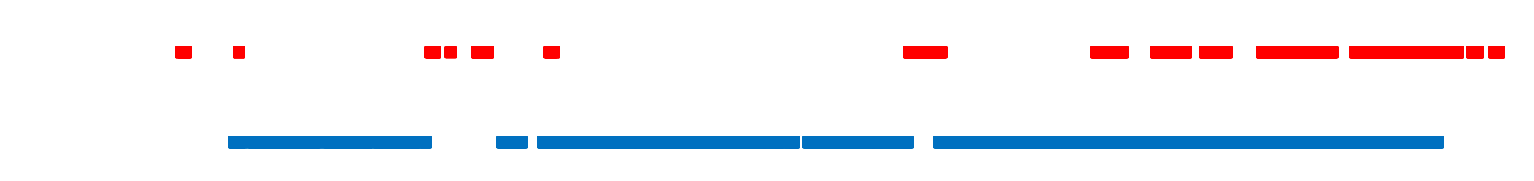


*dipA*


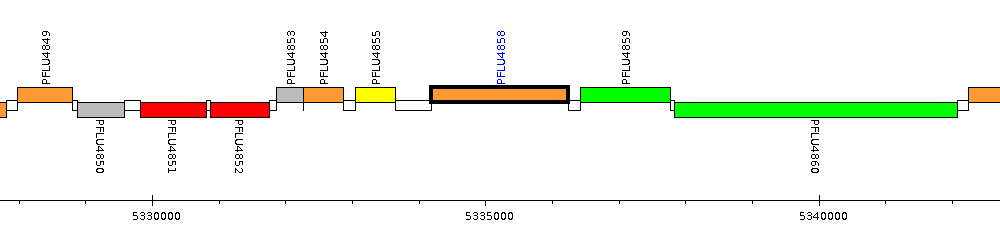

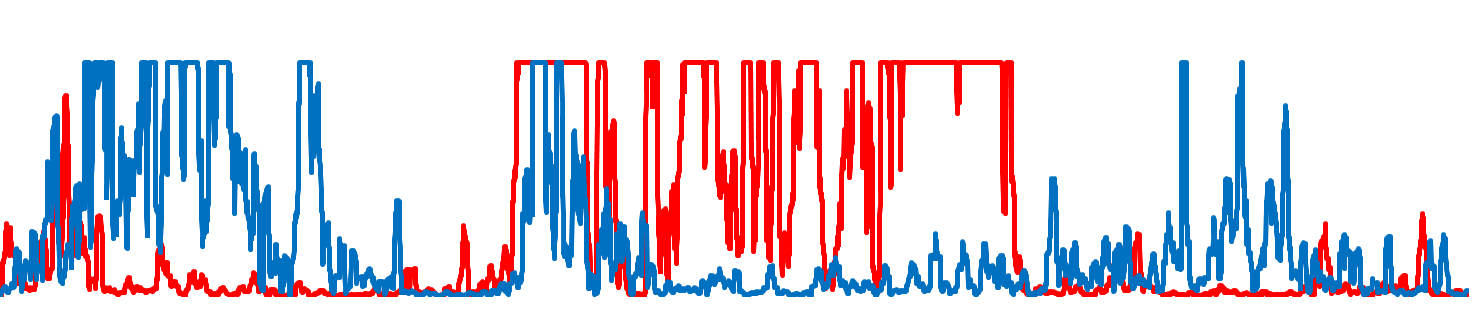

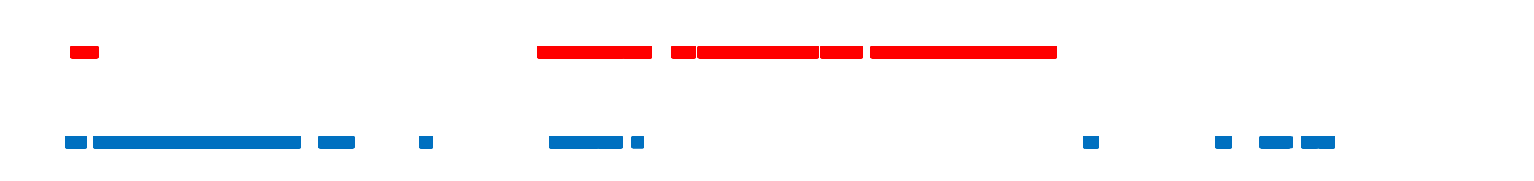


*bifA*


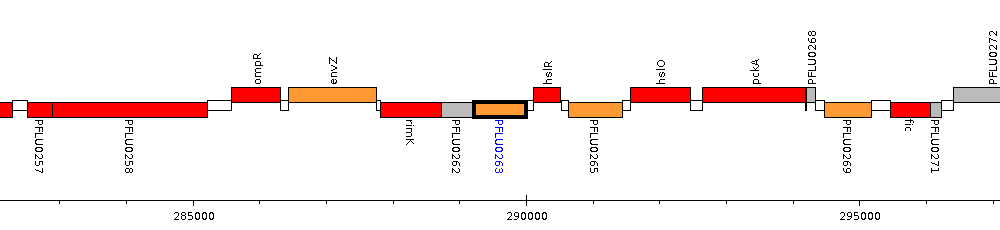

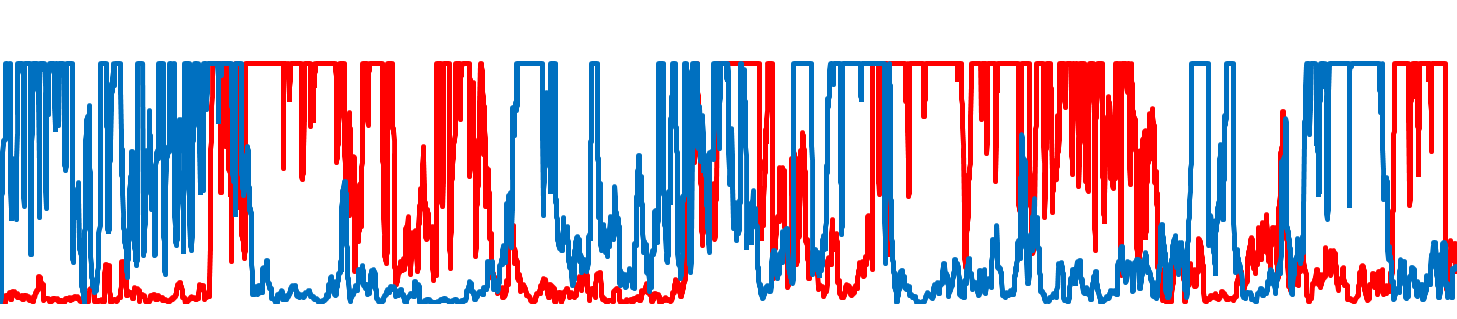

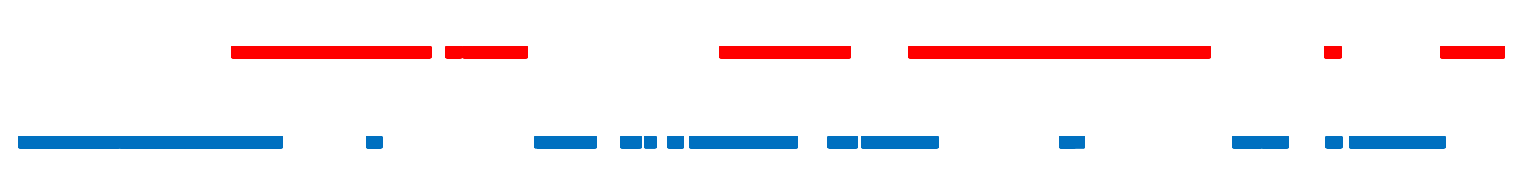


*rimA*


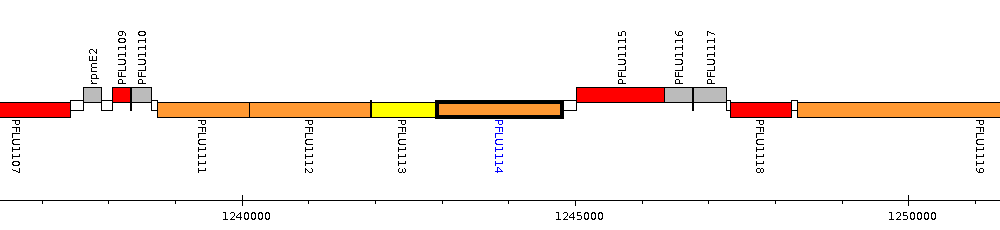

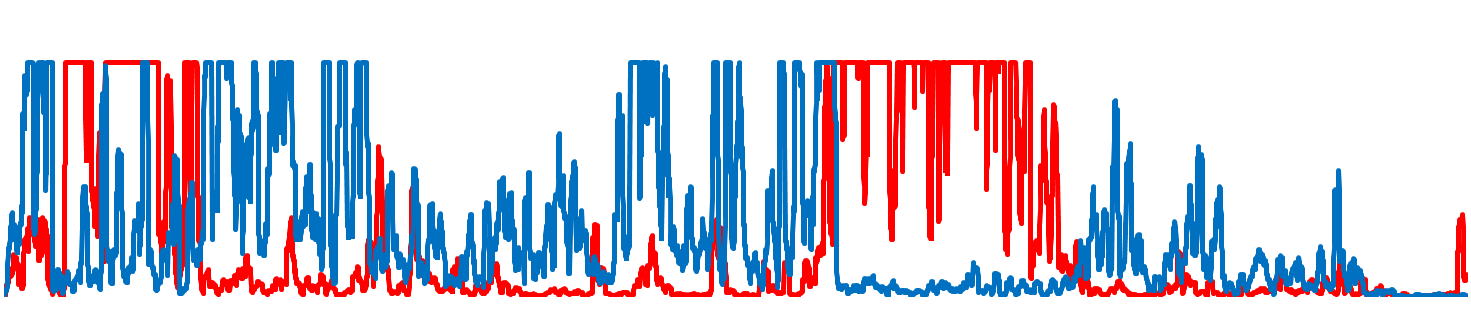

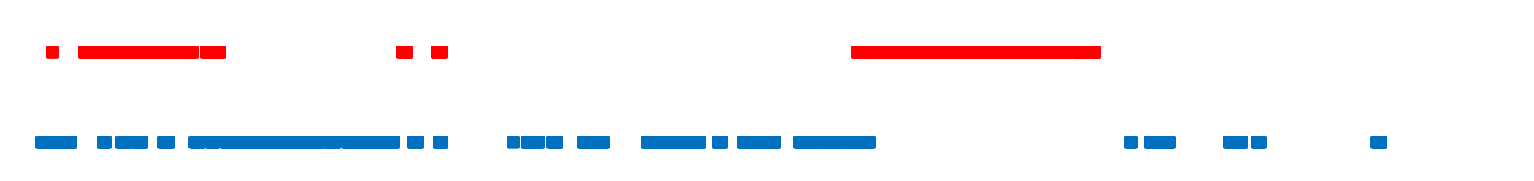


*PFLU1114*

**Supplementary Figure S8A. Transcriptional landscapes of c-di-GMP genes**.


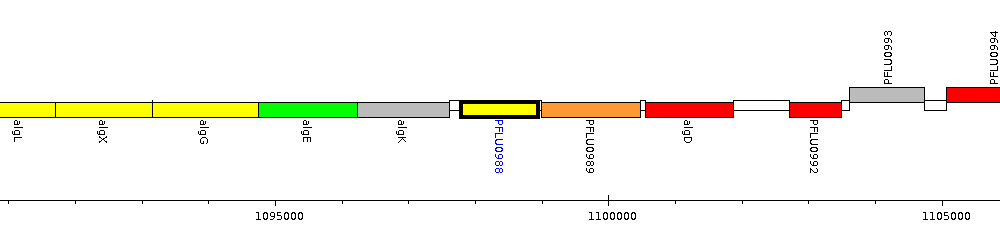

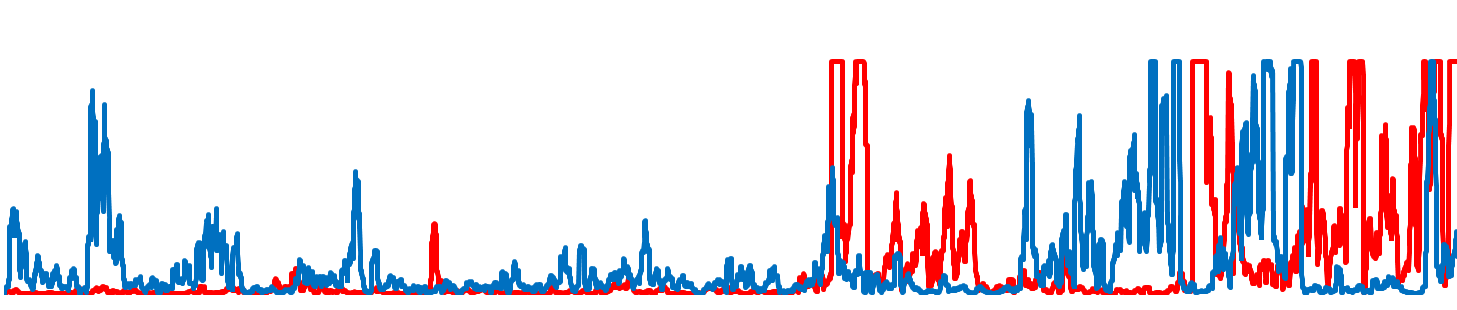

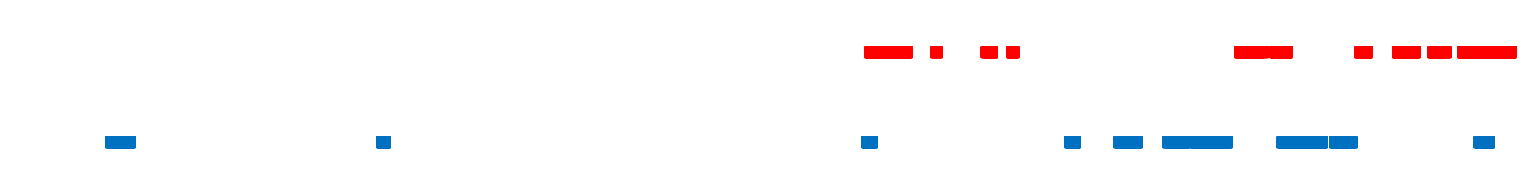


*alg44*


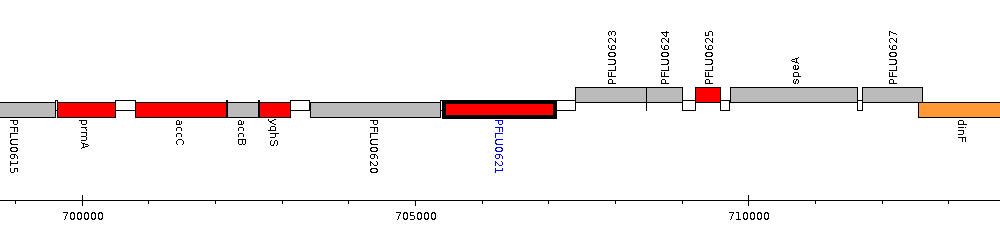

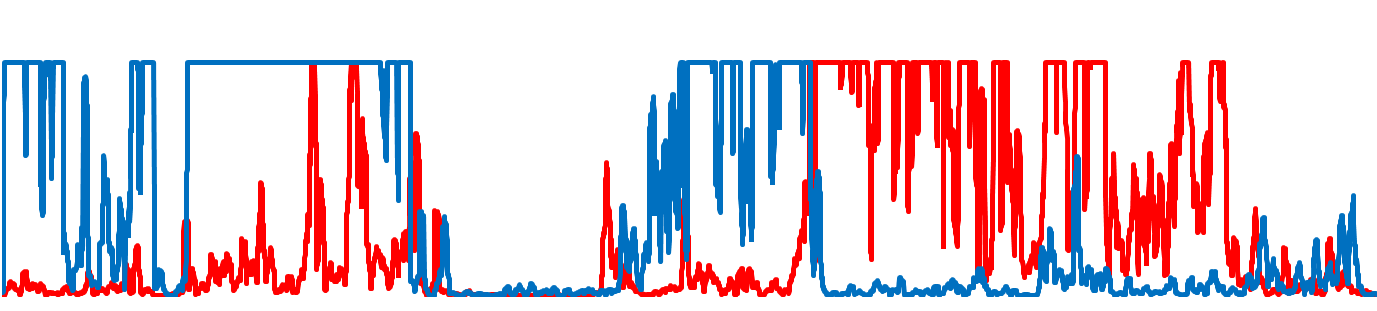

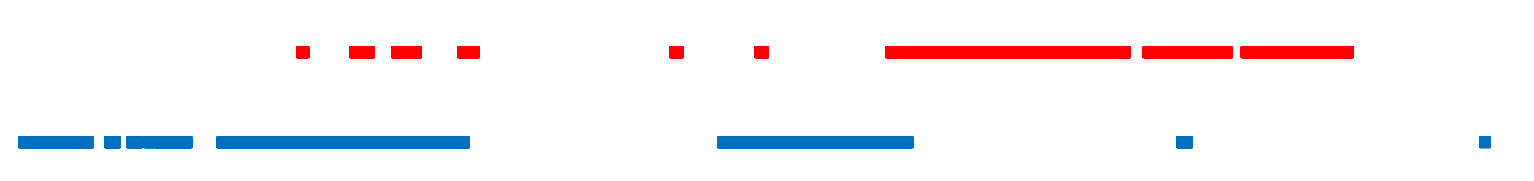


*gcbA*

**Supplementary Figure S8B. Transcriptional landscapes of c-di-GMP genes.**

Transcriptomic data collected from previous experiments^2^ was used to generate gene expression profiles. In these experiments, P. fluorescens SBW25 was cultured in a modified minimal medium. Thirty transcriptomes were combined to generate alignment figures. Alignments are shown over a region of 1400 bp, including equally sized regions up and downstream of gene of interest. In figure, x-axis is chromosomal location and y-axis is number of sequence fragments aligned at that location. Red indicates alignment in the positive orientation and blue indicated alignment in the negative orientation. Line graph indicates total aligned at genomic location, with a maximum number of alignments limited to 500 in figure for clarity. Solid bars indicate regions for which at least 200 alignments in a particular orientation are detected.

2- Larsen, P. E. et al. Modeling the Pseudomonas Sulfur Regulome by Quantifying the Storage and Communication of Information. mSystems 3, doi:10.1128/mSystems.00189-17 (2018).

| gene ID / NCBI | Target gene | Strand | Target sequence |
| --- | --- | --- | --- |
| Pc-mNG | Pc2 | NT | TTCATGAGTTGCCGGTAAAC |
| Pc-mNG | Pc3 | T | GATATACATATGAATTCGAA |
| Pc-mNG | Pc4 | T | CGGTCTGTAGGCTGTAATGC |
| Pc-mNG | Pc5 | NT | TTACAGCCTACAGACCGAGA |
| PFLU_RS04730 | pflu_0952 | T | AGACAACATCCCCGCCAGCC |
| PFLU_RS04731 | pflu_0952 | NT | CACTTTGATGACCGGGCTGG |
| PFLU_RS04305 | pflu_0863 | T | CAGCGATCTTTCCATTGACC |
| PFLU_RS04306 | pflu_0863 | NT | CAGGTCAATGGAAAGATCGC |
| PFLU_RS18410 | pflu_3777 | T | CTGACAAGAATGGGGATAAA |
| PFLU_RS18410 | pflu_3777 | NT | ATAACGTCAGCAACAGTACG |
| PFLU_RS01300 | pflu_0263 | T | CTCGCCCAACCAGCGCTGCG |
| PFLU_RS01300 | pflu_0263 | NT | GGTGAGTGAAAGGGGGAAGT |
| PFLU_RS02265 | pflu_0458 | T | CCAGCCCGATGTCGCCCGAA |
| PFLU_RS02265 | pflu_0458 | NT | GCCGCCATTCGGGCGACATC |
| PFLU_RS04905 | pflu_0988 | NT | TGGACTACGTTGGCATTTAC |
| PFLU_RS23815 | pflu_4858 | T | GGAACTCAAGAACAGCTTGT |
| PFLU_RS23815 | pflu_4858 | NT | GCCGCCATTCGGGCGACATC |
|  | pflu_1114 | T | TATGACCCCGGATTCAGTCA |
|  | pflu_1114 | NT | TGACTGAATCCGGGGTCATA |
| Block sequence backbone | GCTCAGTCCTAGGTATAATACTAGTNNNNNNNNNNNNNNNNNNNNGTTTTAGAGCTAGAAATAGCAAGTTAAAATAAGGCTAGTCCGTTATCAACTTGAAAAAGTGGCACCGAGTCGGTGCTTTTTTTGAAGCTTGGGCCCGAAC | | |

**Table S1: List of gBlocks gene fragments for gRNA designs.**

**Table S2: Quantification of mNG gene silencing by CRISPRi in *P. fluorescens*.**

Fluorescence in cultures of SBW25, WH6 and Pf0-1 cells expressing *Pc-mNG* was measured as the median of the flow cytometry peaks 7 hours after induction of dCas9 by addition of 0.1µg/ml aTc (see Figure S2). Gene silencing can be measured relative to a control which is the same strain without dCas9 induction (non-induced, NI) or relative to a control strain without gRNA but in which dCas9 is induced (no-guide, NG). gRNAs were copied from the non-template (NT) or template (T) DNA strand and targeted transcription initiation or elongation block (Figure S2). Mean values of fluorescence decrease were measured from independent experiments (n ≥ 3) and standard deviation of the means are in parenthesis. ND, not determined.

**Table S3: List of oligonucleotides for generation of gene deletions**

**
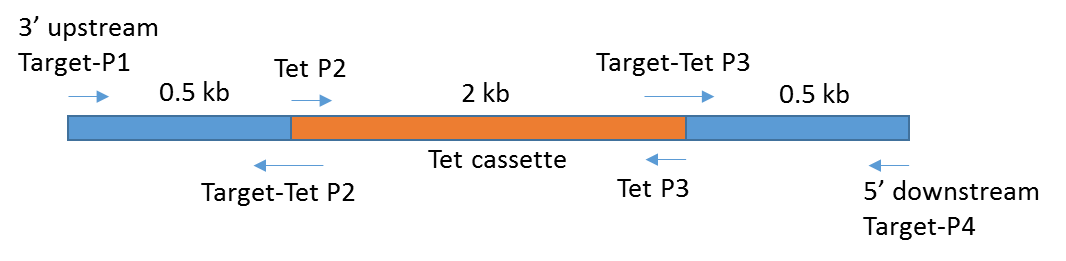
**


**Table S4: Statistical comparison of CRISPRi gene knockdowns and gene knockouts in biofilm phenotypes.**

Air-liquid biofilms were imaged by confocal microscopy (as shown Figure 5). Parameters (Mean Thickness, Maximum thickness ad roughness) were determined using the Biofilm Analysis XTension pack of the Imaris software (Bitplane AG, Zurich, Switzerland). Average and standard deviations were determined over n values (as indicated) obtained from more than 4 independent biofilm pellicles. Fold change and the significance of fold changes for all genes were calculated relative to the control condition using the 2-tailed T-test. A threshold of a P value of less than 0.05 was used for statistical significance determination. Red= statistically significant increase, green= statistically significant decrease relative to control condition (WT strain, no guide RNA).
